# Effects of air pollution on adverse birth outcomes and pregnancy complications in the U.S. state of Kansas (2000–2015)

**DOI:** 10.1038/s41598-023-48329-5

**Published:** 2023-12-06

**Authors:** Hua Hao, Sodahm R. Yoo, Matthew J. Strickland, Lyndsey A. Darrow, Rohan R. D’Souza, Joshua L. Warren, Shannon Moss, Huaqing Wang, Haisu Zhang, Howard H. Chang

**Affiliations:** 1 Gangarosa Department of Environmental Health, Rollins School of Public Health, Emory University, 1518 Clifton Rd., NE, Atlanta, GA 30322 USA; 2 Department of Biostatistics and Bioinformatics, Rollins School of Public Health, Emory University, Atlanta, GA 30322 USA; 3 Depatment of Health Analytics and Biostatistics, Epidemiology and Environmental Health, School of Public Health, University of Nevada, Reno, NV 89557 USA; 4 Department of Biostatistics, School of Medicine, Yale University, New Haven, CT 06510 USA; 5 Department of Landscape Architecture and Environment Planning, College of Agriculture and Applied Sciences, Utah State University, Logan, UT 84322 USA

**Keywords:** Epidemiology, Environmental impact, Preventive medicine

## Abstract

Neonatal mortality and morbidity are often caused by preterm birth and lower birth weight. Gestational diabetes mellitus (GDM) and gestational hypertension (GH) are the most prevalent maternal medical complications during pregnancy. However, evidence on effects of air pollution on adverse birth outcomes and pregnancy complications is mixed. Singleton live births conceived between January 1st, 2000, and December 31st, 2015, and reached at least 27 weeks of pregnancy in Kansas were included in the study. Trimester-specific and total pregnancy exposures to nitrogen dioxide (NO_2_), particulate matter with an aerodynamic diameter less than 2.5 μm (PM_2.5_), and ozone (O_3_) were estimated using spatiotemporal ensemble models and assigned to maternal residential census tracts. Logistic regression, discrete-time survival, and linear models were applied to assess the associations. After adjustment for demographics and socio-economic status (SES) factors, we found increases in the second and third trimesters and total pregnancy O_3_ exposures were significantly linked to preterm birth. Exposure to the second and third trimesters O_3_ was significantly associated with lower birth weight, and exposure to NO_2_ during the first trimester was linked to an increased risk of GDM. O_3_ exposures in the first trimester were connected to an elevated risk of GH. We didn’t observe consistent associations between adverse pregnancy and birth outcomes with PM_2.5_ exposure. Our findings indicate there is a positive link between increased O_3_ exposure during pregnancy and a higher risk of preterm birth, GH, and decreased birth weight. Our work supports limiting population exposure to air pollution, which may lower the likelihood of adverse birth and pregnancy outcomes.

## Introduction

Preterm birth and low birth weight are leading causes of neonatal morbidity and mortality, and also associate with increased morbidity in adulthood^1,2^. In 2020, the prevalence of preterm birth in the United States (U.S.) was 10.09%, and that of low birth weight was 8.24%^3^. Preterm birth complications range from early impacts on the digestive, respiratory, and central nervous systems to late consequences on cognitive, motor, auditory, visual, behavioral, and social-emotional function, as well as a variety of implications for growth and health^4^. The financial burden linked to preterm birth in the U.S. in 2016 was estimated to be on average $76,153 per infant, and low birth weight (< 2500 g) was associated with average expenses of $114,437^5^. Previous epidemiological studies have suggested that ambient air pollution may increase the risks of preterm birth and low birth weight, although the evidence remains inconsistent across different pollutants and exposure windows^1,6,7^. Variations in epidemiologic modeling strategies and exposure assessment methods between research may contribute somewhat to these discrepancies^8^.

Gestational diabetes mellitus (GDM) and gestational hypertension (GH) are the most prevalent maternal medical complications during pregnancy^9^. Previous research has shown that developing diabetes before the 24th week of pregnancy increases the risk of preterm birth^10^, and that women with pregnancy-induced hypertension had a 3.89-fold increased (95% CI: 2.66, 5.69) chance of delivering low birth weight newborns^11^. GDM prevalence varies between 5.8 and 12.9%^12^, and a previous study reported that 7.1–9.2% of GDM mothers would develop type 2 diabetes within 5 years^13^. In the U.S., GH complicates up to 10% of pregnancies^14^ and the prevalence of GDM and GH is increasing globally. Both are related to short- and long-term health risks and a significant burden on maternal and child health, including the risk for cardiovascular and cardiometabolic disorders development in later life^15,16^. Previous epidemiological research has investigated the connections between air pollution and pregnancy complications (including GDM and GH)^17–20^. However, because of differences in population, study design, exposure measurement, and GDM diagnosis, the links between air pollution and pregnancy complications have conflicting results and are less conclusive.

In this study, we investigated the connections between three ambient air pollutants of nitrogen dioxide (NO_2_), particulate matter with an aerodynamic diameter less than 2.5 μm (PM_2.5_), and ozone (O_3_), and two birth outcomes (preterm birth and birth weight) and two pregnancy complications (GDM and GH) in the State of Kansas. We also presented the effect modification of those associations by maternal race, maternal ethnicity, maternal age, and maternal residual address zip code level percent below poverty. To the best of our knowledge, this is the first epidemiologic research on air pollution and pregnancy outcomes in Kansas.

## Methods

### Data and outcome assessments

**Figure 1:**
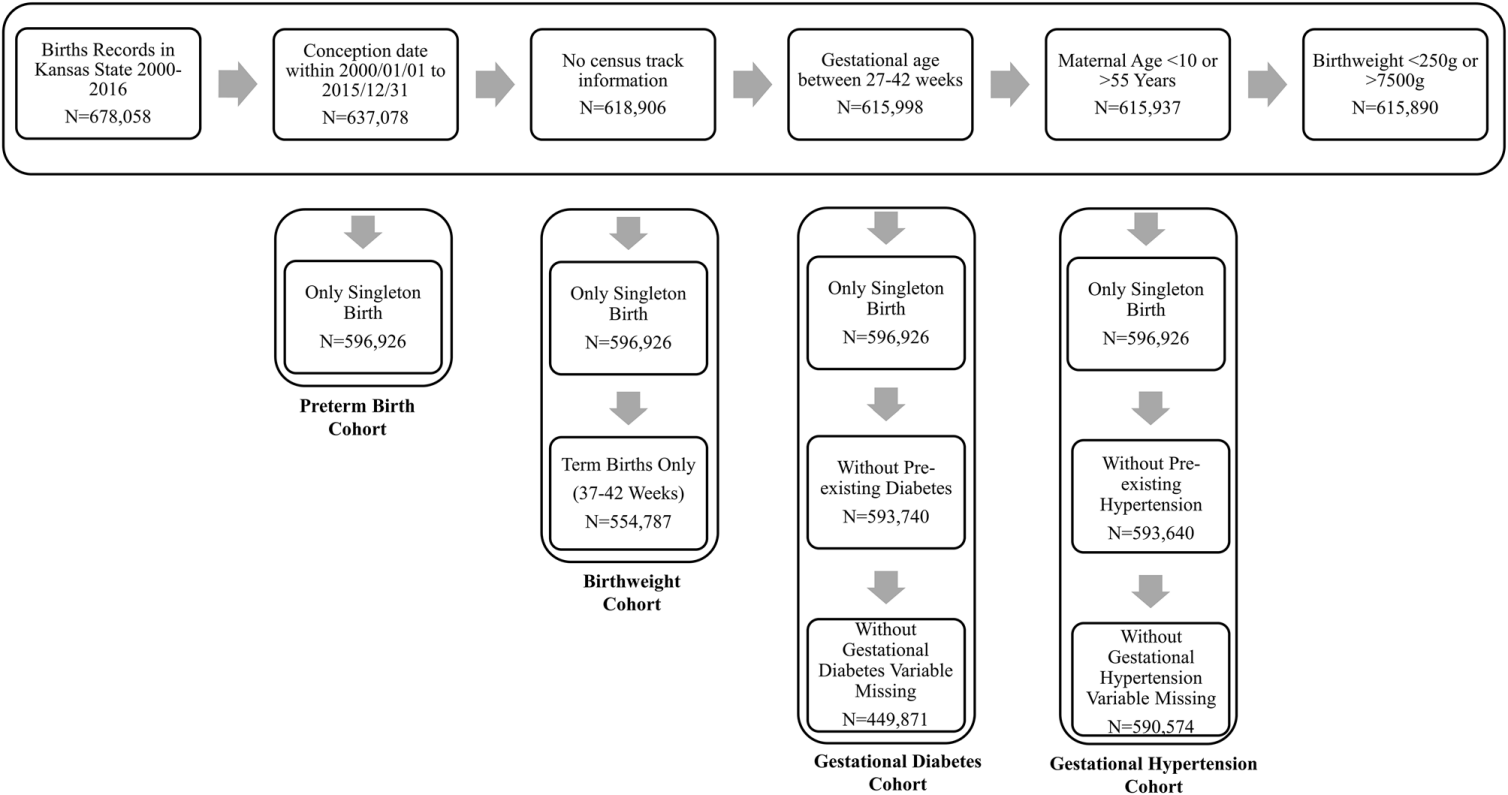
Study inclusion and exclusion criteria for analysis of air pollution exposure and adverse birth outcomes and pregnancy complications in the U.S. state of Kansas between January 1st, 2000 and December 31st, 2015.

Birth certificate data were obtained from the Bureau of Epidemiology and Public Health Informatics, Kansas Department of Health for the period 1999–2016. Inform consent was not obtained or required because this study relies on secondary data analysis of registry data, and no contact with participants were initiated. The study was approved by the Institutional Review Board of the Kansas Department of Health. We confirm that informed consent was obtained from all subjects and/or their legal guardian(s) by the Kansas Department of Health. To minimize fixed-cohort bias and establish a cohort defined by conception rather than birth date with constant gestational age distributions over calendar time^21^, only singleton pregnancies with a gestational age of 27–42 weeks with an estimated date of conception between January 1st, 2000 and December 31st, 2015, were included in our analysis. The date of the last menstrual cycle was utilized to determine gestational age, and 14 days was added to that date to estimate the date of conception. By only including births ≥ 27 weeks, we ensured that all births were followed for the entirety of the first (gestational weeks 1–13) and second trimesters (gestational weeks 14–26). The birth outcomes of interest were preterm birth (gestational age < 37 weeks), term (gestational age ≥ 37 weeks) birth weight, GDM (self-reported), and GH (self-reported). We excluded births with (a) missing census tract information; (b) maternal age < 10 years or > 55 years; (c) birth weight < 250 g or > 7500 g. For the outcomes of GDM and GH, we also excluded mothers with pre-existing diabetes or pre-existing hypertension. Figure 1 describes the number of births retained after applying each exclusion criterion. After exclusions, 596,926 and 554,787 births were eligible for the preterm birth and birth weight analysis, respectively, and 449,871 and 590,574 pregnancies were eligible for the GDM and GH analysis, respectively.

### Exposure assessment

Employing spatiotemporal ensemble models, daily ambient air pollution data for NO_2_ (1-h maximum), PM_2.5_ (24-h average), and O_3_ (8-h maximum) concentrations were calculated at a 1 km × 1 km spatial resolution across the United States from 2000 to 2016^22^. In particular, the ensemble-based models utilized over one hundred predictors and incorporated three machine learning algorithms (including random forest, a neural network, and gradient boosting machine) with good prediction performance^23^. For each day, air pollution data were averaged to the at-delivery census tract of the mother by averaging the daily concentrations of all 1 km^2^ grid cells whose centroids were within the census tract boundary.

### Statistical analysis

We examined associations between air pollution concentrations and birth outcomes in single-pollutant models for each exposure window of interest for all analyses. We applied logistic regression to determine relationships between preterm birth (< 37 weeks of gestation) and mean air pollution concentrations during the first trimester (weeks 1–13 of gestation), and second trimester (weeks 14–26 of gestation), and a discrete-time survival model to assess relationships during the third trimester (pregnancy week 27 to the end of week *t*) and total pregnancy (pregnancy week 1 to the end of week *t*) to avoid bias because of varying exposure periods between preterm and full-term births^24^. Linear regression models were used to examine term birth weight as a continuous outcome and mean air pollution concentrations for the first trimester, second trimester, third trimester, and total pregnancy exposure windows. For GDM, we utilized logistic regression to assess the connections between GDM and averaged pollutant concentrations during the first trimester, and second trimester because screening for GDM is typically completed between 24 and 28 weeks of gestation^25^. Logistic regression models were utilized to evaluate the relationship between GH and averaged pollutant levels during the first trimester, second trimester, third trimester, and total pregnancy exposure windows.

For all four outcomes, we controlled for the following covariates: maternal characteristics included education level (< High school, High school or equivalent, Some college/Associate degree, Bachelor’s or above), race (White, African American, Asian, Other), maternal ethnicity (Hispanic, Non-Hispanic), maternal age modeled employing natural cubic spline with 3 degrees of freedom, marital status (Married, Unmarried, Missing), birth parity (1, 2, ≥ 3), and smoking status during pregnancy (Yes or No). Temporal controls included the season of conception (Winter: December–February; Spring: March–May; Summer: June–August; Fall: September–November) and a natural cubic spline with 5 degrees of freedom on the conception date used for a long-term temporal trend. Areal-level covariates included ZIP code-level percent below poverty information from the 2010 Census. Census tract-level land area and water surface area (square meters) as well as percent of greenspace, both were calculated from the National Land Cover Database (NLCD) of the United States Geological Survey. Finally, we included indicators for each of the 106 maternal residential counties in all models.

We stratified the data by maternal race (White, African American, Asian, other), maternal ethnicity (Hispanic versus Non-Hispanic), maternal age (27 years versus > 27 years), and maternal residential address zip code level percent below poverty (12% versus > 12%), with different regression models apply to each stratum, in order to analyze effect modification of the relationship between air pollution and each birth outcome.

Several sensitivity analyses were performed to assess the validity of our key findings. First, we fitted the model by adjusting the long-term temporal trend using a natural cubic spline with 8 and 10 degrees of freedom on the conception date, or adjusted the conception year as a categorical variable. Second, since labor inductions are typically medically indicated, we further restricted to births without maternal induction of labor. Third, to control the confounding by exposures in other windows^26^, we run a single model to jointly estimate the association with each of the three trimester-average exposures (trimester 1, trimester 2, and trimester 3) for outcomes of preterm birth, birthweight, and GH, and two trimester-average exposures (trimester 1 and trimester 2) for the outcome of GDM. Fourth, we fitted multi-pollutant models by including trimester specific NO_2_, PM_2.5_, and O_3_ in the model simultaneously.

We report odds ratios (ORs) and mean differences for each interquartile range (IQR) increment in air pollution concentrations. Average pollutant levels during pregnancy were utilized to compute the IQRs, and we applied the same IQR values derived from the preterm birth cohort to report results from the birth weight, GDM, and GH outcomes. R software, version 4.0.2, was employed to perform all statistical analyses and all methods were performed in accordance with the relevant guidelines and regulations.

### Ethics approval and consent to participate

Human subjects research approved by Emory University IRB #00102330.

## Results

### Study population characteristics

The preterm birth cohort comprised 596,926 singleton births across 106 Kansas counties, of which 41,936 (7.0%) were preterm. Compared to full-term birth mothers, preterm birth mothers had a high chance to deliver low birth weight babies, have gestational diabetes and chronic diabetes, have gestational hypertension and chronic hypertension, have lower education level, be of younger age, be unmarried, have higher parity, smoke during pregnancy, and live in more impoverished zip code area (Table 1). During the study period, the statewide incidence of preterm birth increased slightly (from 6.7% in 2000 to 7.0% in 2015). Characteristics of the birth weight, GDM, and GH cohorts were presented in Table S1.

**Table 1:** Maternal and infant characteristics from birth records of preterm and full-term singleton births in Kansas with an estimated date of conception from January 1st, 2000, to December 31st, 2015.

Maternal/infant characteristics	Preterm birth (N = 41,936)	Full-term birth (N = 554,990)	Total (N = 596,926)
Birth weight (g)			
Mean (SD)	2499.5 (644.6)	3410.9 (458.5)	3346.9 (528.1)
Gestational diabetes			
Yes	2095 (5.0)	18,360 (3.3)	20,455 (3.4)
No	29,584 (70.5)	403,017 (72.6)	432,601 (72.5)
Missing	10,257 (24.5)	133,613 (24.1)	143,870 (24.1)
Chronic diabetes			
Yes	720 (1.7)	2466 (0.4)	3186 (0.5)
No	30,959 (73.8)	418,912 (75.5)	449,871 (75.4)
Missing	10,257 (24.5)	133,612 (24.1)	143,869 (24.1)
Gestational hypertension			
Yes	5,024 (12.0)	19,234 (3.5)	24,258 (4.1)
No	36,907 (88.0)	535,695 (96.5)	572,602 (95.9)
Missing	5 (0.0)	61 (0.0)	66 (0.0)
Chronic hypertension			
Yes	1127 (2.7)	5159 (0.9)	6286 (1.1)
No	40,804 (97.3)	549,770 (99.1)	590,574 (98.9)
Missing	5 (0.0)	61 (0.0)	66 (0.0)
Gestational age (weeks)			
Mean (SD)	34.5 (2.1)	39.1 (1.1)	38.76 (1.66)
Maternal education level			
< High School	8114 (19.5)	91,530 (16.6)	99,644 (16.8)
High School/GED	11,659 (28.0)	136,321 (24.7)	147,980 (24.9)
Some College/AA	12,023 (28.8)	159,097 (28.8)	171,120 (28.8)
BA+	9890 (23.7)	165,201 (29.9)	175,091 (29.5)
Maternal ethnicity			
Hispanic	11,471 (27.5)	137,984 (25.0)	149,455 (25.1)
Non-Hispanic	30,465 (72.5)	417,006 (75.0)	447,471 (74.9)
Maternal age (years)			
Mean (SD)	26.9 (6.1)	27.0 (5.8)	27.0 (5.8)
Maternal marital status			
Married	18,186 (43.4)	268,924 (48.5)	287,110 (48.1)
Unmarried	13,458 (32.1)	152,167 (27.4)	165,625 (27.7)
Missing	10,292 (24.5)	133,899 (24.1)	144,191 (24.2)
Parity			
1	11,650 (45.8)	175,844 (50.7)	187,494 (50.3)
2	7293 (28.7)	100,957 (29.1)	108,250 (29.1)
≥ 3	6507 (25.6)	70,325 (20.3)	76,832 (20.6)
Smoking during pregnancy			
Yes	5094 (12.1)	48,670 (8.8)	53,764 (9.0)
No	36,842 (87.9)	506,320 (91.2)	543,162 (91.0)
Conception season			
Winter	10,425 (24.9)	137,994 (24.9)	148,419 (24.9)
Spring	10,548 (25.2)	136,057 (24.5)	146,605 (24.6)
Summer	10,450 (24.9)	137,268 (24.7)	147,718 (24.7)
Autumn	10,513 (25.1)	143,671 (25.9)	154,184 (25.8)
Zip code level percent below poverty			
Mean (SD)	13.0% (7.0%)	12.0% (7.0%)	12.0% (7.0%)
Census tract level percentage of greenspace			
Mean (SD)	58.0 (33.7)	57.7 (33.5)	57.7 (33.5)

### Air pollution levels

**Figure 2:**
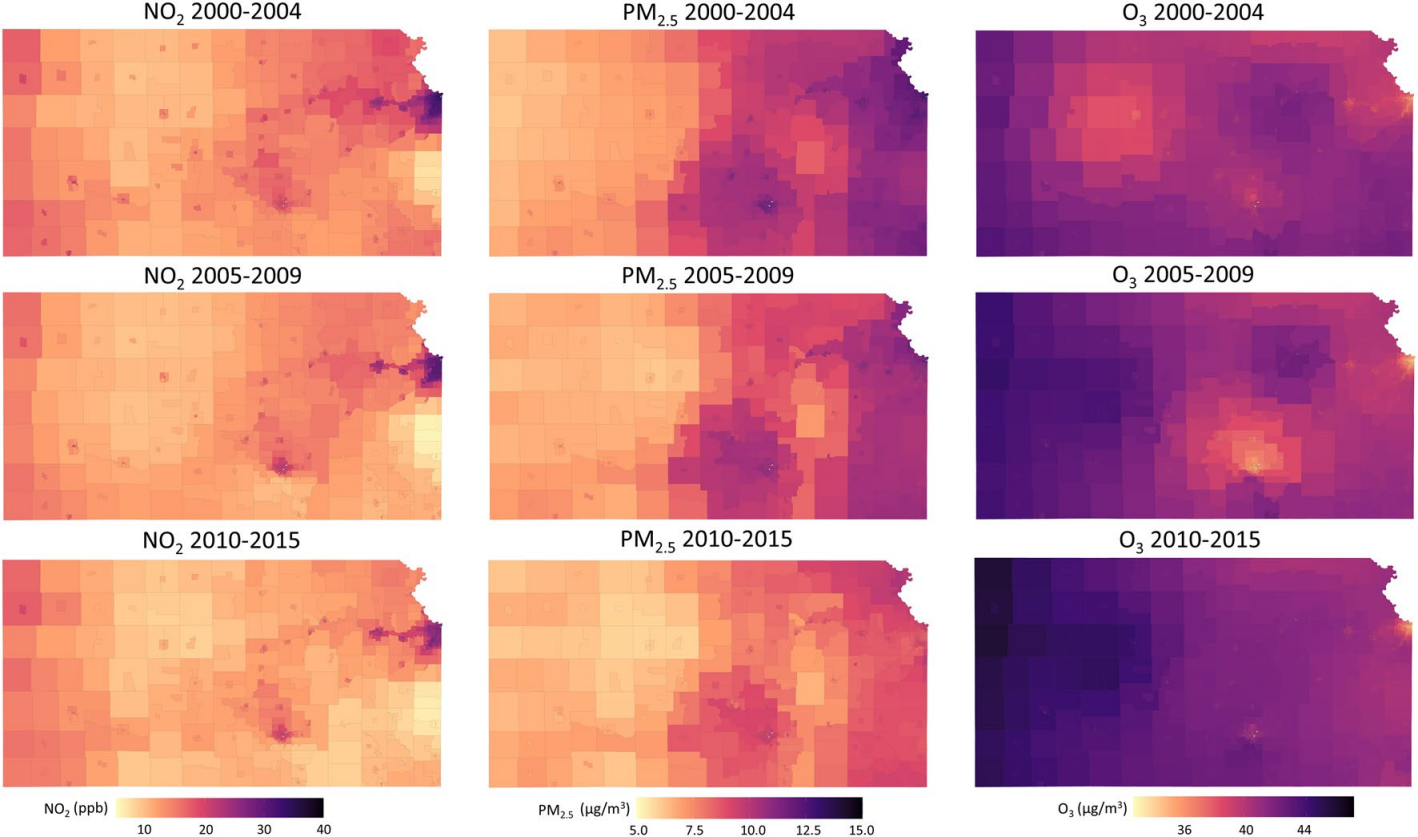
Average concentrations of NO_2_, PM_2.5_, and O_3_ over the Kansas state from 2000 to 2015 (from upper row to the lower row represent the average concentrations during 2000–2004, 2005–2009, 2010–2015 for three pollutants) as estimated by using spatiotemporal ensemble models, which utilized over one hundred predictors, integrated three machine learning algorithms, and achieved excellent model performance.

The census tract-level spatial distributions of three air pollutants (NO_2_, PM_2.5_, O_3_) in Kansas during the sixteen-year study period are presented in Fig. 2. Average NO_2_ and PM_2.5_ decreased from 2000–2004 to 2010–2015 (NO_2_: 12.12 to 10.23 ppb; PM_2.5_: 9.01 to 7.72 µg/m^3^), but the daily mean of 8-h maximum O_3_ increased from 40.82 to 42.43 µg/m^3^ during the same periods. The interquartile range (IQR) of the of specific pollutants and pregnancy exposure window pairs are: first trimester NO_2_: 10.90 (ppb); second trimester NO_2_: 10.88 (ppb); third trimester NO_2_: 11.01 (ppb); total pregnancy NO_2_: 10.16 (ppb); first trimester PM_2.5_: 2.86 (µg/m^3^); second trimester PM_2.5_: 2.89 (µg/m^3^); third trimester PM_2.5_: 3.04 (µg/m^3^); total pregnancy PM_2.5_: 2.86 (µg/m^3^); first trimester O_3_: 13.82 (µg/m^3^); second trimester O_3_: 13.51 (µg/m^3^); third trimester O_3_: 14.26 (µg/m^3^); total pregnancy O_3_: 6.23 (µg/m^3^) (Table S2). Figure S1 shows the correlation matrix of three air pollutants (NO_2_, PM_2.5_, O_3_), and Fig. S2 shows the correlation matrix between different exposure windows for NO_2_, PM_2.5_, and O_3_. Correlations between pollutants in the same exposure window were low to moderate (< 0.45), while correlations between trimesters of the same pollutant can be high due to its seasonality.

### Health effect estimates

**Figure 3:**
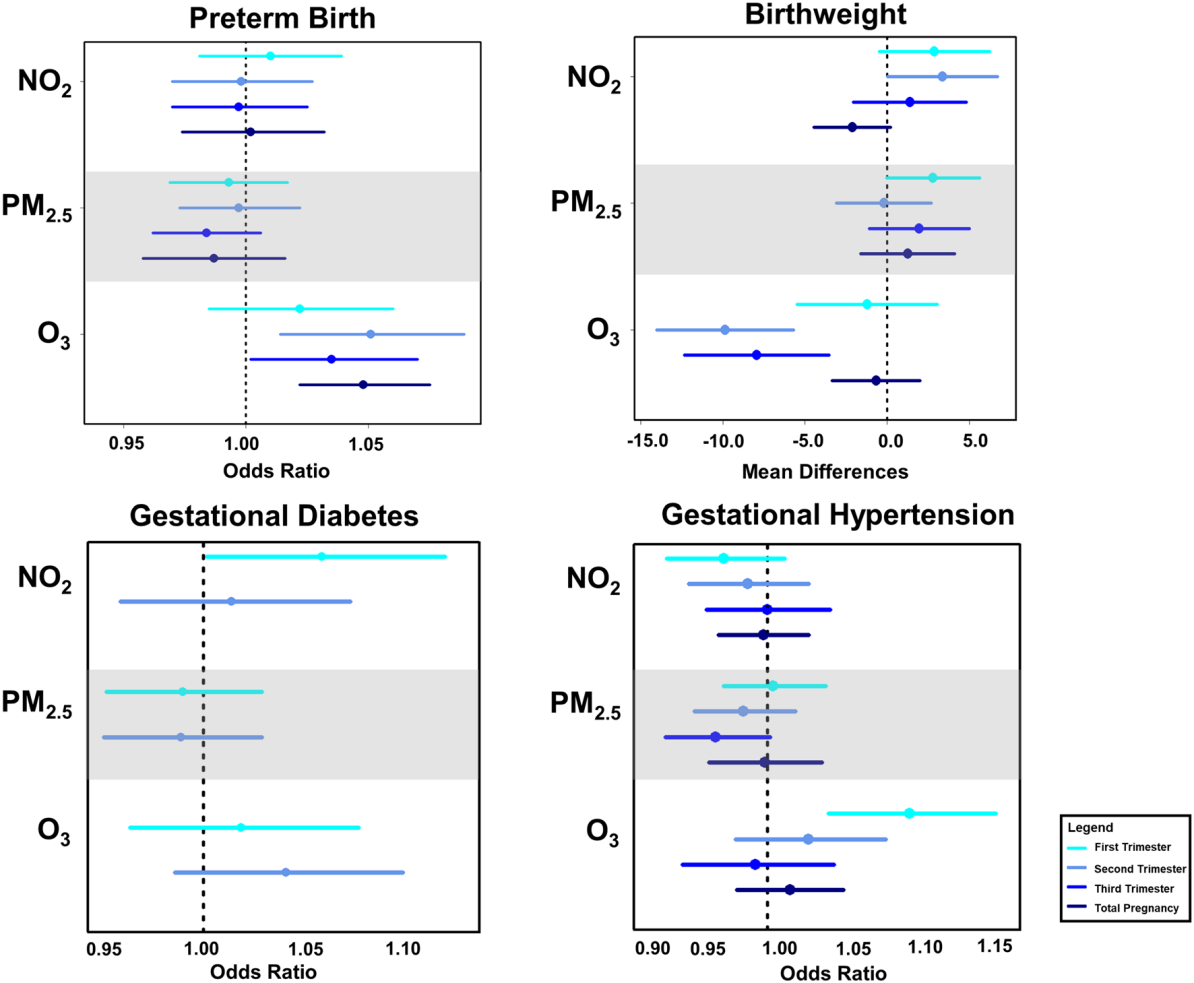
Statewide trimester-specific and total pregnancy-adjusted odds ratios (ORs) and mean difference and 95% confidence intervals per interquartile range (IQR) increase in the concentration of 3 pollutants. Models were adjusted for maternal education level, race, ethnicity, age, marital status, smoking, infant parity, zip code level percent below poverty, census tract level greenness, season of conception, and long-term trend using a natural cubic spline on conception date with 5 degrees of freedom. IQRs: First 4 Weeks NO_2_: 11.26 (ppb); First 6 Weeks NO_2_: 11.16 (ppb); First Trimester NO_2_: 10.90 (ppb); Second Trimester NO_2_: 10.88 (ppb); Third Trimester NO_2_: 11.01 (ppb); Total Pregnancy NO_2_: 10.16 (ppb); First 4 Weeks PM_2.5_: 3.56 (µg/m^3^); First 6 Weeks PM_2.5_: 3.30 (µg/m^3^); First Trimester PM_2.5_: 2.86 (µg/m^3^); Second Trimester PM_2.5_: 2.89 (µg/m^3^); Third Trimester PM_2.5_: 3.04 (µg/m^3^); Total Pregnancy PM_2.5_: 2.86 (µg/m^3^); First 4 Weeks O_3_: 15.81 (µg/m^3^); First 6 Weeks O_3_: 15.56 (µg/m^3^); First Trimester O_3_: 13.82 (µg/m^3^); Second Trimester O_3_: 13.51 (µg/m^3^); Third Trimester O_3_: 14.26 (µg/m^3^); Total Pregnancy O_3_: 6.23 (µg/m^3^).

Figure 3 presents the statewide estimated relationships of exposure to three pollutants (NO_2_, PM_2.5_, O_3_) on preterm birth, birth weight, GDM, and GH during different pregnancy exposure windows. We identified elevated risks of preterm birth with IQR increment in second trimester O_3_ (odds ratio [OR]: 1.05; 95% confidence interval [CI]: 1.01, 1.09), third trimester O_3_ (OR: 1.04; 95% CI: 1.00, 1.08), and total pregnancy O_3_ (OR: 1.05; 95% CI: 1.02, 1.08). Per IQR increase in the second and third trimester O_3_, mean difference in term birth weight were − 9.86 (95% CI: − 14.00, − 5.69) and − 7.93 (− 12.34, − 3.53) grams. Second-trimester exposure to NO_2_ was positively related to the risk of GDM (OR: 1.06; 95% CI: 1.00, 1.12). O_3_ in the first trimester (T1) of pregnancy were connected to increased risk of GH (T1: 1.11; 95% CI: 1.05, 1.17). We didn’t observe any associations related to PM_2.5_ exposure. Effect estimates and 95% CI for all outcomes, pollutants and exposures are given in Table S3.

### Effect modifications

Table S4 shows subgroup-specific results stratified by maternal race, maternal ethnicity, maternal age, and maternal residential address zip code level percent below poverty. We did not observe consistent results across all outcomes and pollutants. However, mothers living in higher ZIP code level percent below poverty tend to have worse birth outcomes and pregnancy complications related to air pollution exposure. For example, the associations between NO_2_ and preterm birth are 0.98 (95% CI: 0.94, 1.02) and 1.04 (95% CI: 1.00, 1.08) for mothers living in ZIP codes with lower versus higher percent below poverty, respectively.

### Sensitivity analysis

Our findings were robust to a number of sensitivity analyses. First, we examined the sensitivity of results to the approach for adjusting for long-term trend by fitting the model using a spline with 8 and 10 degrees of freedom on the conception date, or adjusting the conception year as a categorical variable. The estimated effects remained largely stable compared to the main analysis (Table S5). Second, we observed consistency in our preterm birth results after excluding births with induced labor (Table S6). Third, in comparison to the primary analysis, the estimated effects stayed consistent after including all related trimester-average exposures in the model (Table S7). Fourth, under multi-pollutant models, we observed consistency in our results (Table S8).

## Discussion

This retrospective cohort study aimed to examine the relationship between ambient air pollution (NO_2_, PM_2.5_, O_3_) exposure during pregnancy and the risks of adverse birth outcomes (preterm birth and birth weight) and pregnancy complications (GDM and GH) using spatially-resolved exposure assessment methods. Our findings indicate that higher O_3_ exposure during pregnancy elevates the risk of preterm birth, lower birth weight, and gestational hypertension. We also observed that early-pregnancy NO_2_ exposure elevated the risk of GDM.

Our observation that exposure to O_3_ was positively connected to the risk of preterm birth is similar with prior studies. Previous research from various parts of the world have discovered that O_3_ exposures raise the risk of preterm birth^27,28^. A recent systematic review of 15 studies across 4 continents (Australia, Asia, Europe, and North America) reported that the pooled effect per 10 µg/m^3^ increment in second trimester exposure to O_3_ was 1.05 (95% CI: 1.02, 1.08)^28^. This finding is consistent with our results—an elevated 5% risk during the second trimester per IQR of 13.51 µg/m^3^. For the associations of whole-pregnancy O_3_ exposure and preterm birth, one previous paper also reported a 3% higher risk of birth before 37 weeks and a 13% enhanced risk of preterm birth deliveries per IQR of 7.1 µg/m^3^^29^. The biological mechanisms underlying the link between O_3_ exposure and preterm birth are primarily mediated by oxidative stress and systemic inflammation^30^. Animal models have shown that O_3_ exposure alters the circulating serum cytokines, which may affect normal placentation, uterine artery vascularity, impaired trophoblast invasion, migration, and decreased trophoblast metabolic capability^30^. These biological mechanisms establish a rational explanation for how exposure to O_3_ during pregnancy might trigger preterm birth.

We detected a negative relationship between O_3_ exposure during the second and third trimesters and term birth weight. Our results are generally consistent with earlier research^31–34^. For example, one previous study discovered that the OR of low birth weight for each IQR increase in ozone concentration was 1.04 (95% CI: 1.02, 1.05) in a California population^31^. However, some previous research reported no relationship between exposure to O_3_ and birth weight^1^. These inconsistent results could be attributed to the various study designs, diverse study populations, and various levels of ozone exposure. Our results suggested significant effect of O_3_ exposure in the second and third trimester on birth weight in the trimester-specific analyses. This finding partially matched the previous results. According to earlier research, the first and third trimesters may be the most vulnerable time period for exposure to adverse birth outcomes^35,36^. However, Salam et al.^34^ reported a link between O_3_ exposure in the second and third trimesters and decreased birth weight which is consistent with our birth weight results. The underlying reasons for the differences remained unknown. Still, to assess the accurate effect of O_3_ exposure on birth weight, additional studies using various epidemiological approaches, different geographic locations, and more participants are needed. The possible underlying biological mechanisms by which O_3_ exposure may lead to reduced birth weight, including inflammatory reactions, blood flow, placental development and function, and oxidative stress^37,38^. Additionally, because pregnancy is characterized by organ formation, high levels of cell proliferation, and changing metabolic capacity, pregnant mothers are more susceptible to the impacts of air pollution^39,40^.

We discovered significant relationship between first trimester NO_2_ exposure and GDM. Previous studies reported inconsistent associations between NO_2_ and GDM. For example, a birth cohort of 256, 372 subjects in New York City found NO_2_ exposure in the first trimester was significantly linked to higher odds of GDM (OR: 1.05 per 7.96 ppb, 95% CI: 1.01, 1.09)^41^. But another study from Southern California with 239,574 subjects reported a weak but not statistically significant relationship between NO_2_ first trimester exposure and GDM (OR: 1.02 per 10.40 ppb, 95% CI: 0.99, 1.05)^18^. The heterogeneity between studies is likely influenced by differences in exposure assessment approaches, or differences in outcome assessment, medical care, population characteristics, or pollution mixtures. Our results also show significant negative associations between early pregnancy O_3_ exposure and GH. One previous study reported that O_3_ exposure during the first two trimester is associated with hypertension during pregnancy (HDP), and early pregnancy is a critical window of exposure^42^. Multiple biological pathways have been proposed to underlie O_3_ and HDP. Hypertension is linked to physiologic reactions to environmental variables, according to previous animal and toxicological research^43^. Pregnant women have been demonstrated to have higher levels of oxidative stress, and inflammation when exposed to air pollution^43,44^. These inflammatory reactions may also result in endothelial dysfunction, autonomic instability, and altered blood rheology, all of which have been associated to an elevated risk of hypertension^45,46^.

We didn’t obverse consistent associations between adverse pregnancy and birth outcomes with PM_2.5_ exposure. Previous meta-analysis studies suggested consistent associations between ambient PM_2.5_ and reduced birth weight, especially the effect estimates based on the entire pregnancy exposure^1^. However, the associations between PM_2.5_ and preterm birth^1^, GDM^47^, and GH^48^ are inconsistent. For example, Tang et al.^47^ did a meta-analysis study based on 1,547,154 individuals, and they concluded increased exposure to PM_2.5_ was not associated with the increased risk of GDM (adjusted OR: 1.03, 95% CI: 0.99, 1.06). The possible explanations for our results include Kansas is a relatively underdeveloped state in the U.S., and it has many rural areas compared to other states, so the PM_2.5_ components of black carbon and sulfate should be relatively lower compared to states with more urban areas. Black carbon and sulfate have been linked to several adverse birth and pregnancy outcomes^49,50^.

Our study has several strengths. First, our analysis was based on a large population of over 500,000 births, and exposure assessment using spatiotemporal ensemble models with high temporal and spatial resolutions^23^, which provided complete coverage over the entire Kansas state geographic region. This approach differs from previous methods that were limited to air quality metrics from only stationary monitors, which lack spatial and temporal resolution. Second, we used a time-to-event framework to model the effect of the third trimester and total pregnancy exposure to air pollution on the risk of preterm birth^24^. This design enables us to evaluate risk for third trimester effectively by minimizing the potential bias brought on by variations in the length of third trimester averaging period between preterm and full-term births^51^. Last, the Kansas birth records contain comprehensive maternal information, which removes the potential recall bias associated with gestational age based on the last menstrual period^52^.

Our research also has a few limitations. First, maternal address at birth is the only address available in our birth certificate data. However, some women may have resided at a different address during pregnancy. This may result in nondifferential exposure misclassification, which might bias the results toward the null. Previous research reported exposure misclassification due to pregnant women’s home address change was limited because most pregnant women did not move throughout their pregnancy^53,54^. Additionally, among mothers who moved, they remained close enough to their previous home address. Therefore, mobility is unlikely to have significantly impacted our findings^55^. Second, GDM and GH were identified through medical records, is subject to outcome misclassification. According to a previous study^56^, GDM misclassification using birth certificate data would lead to an underestimation of the GDM prevalence and further underestimate the relative risk. Third, while we controlled for multiple risk factors in our models, including maternal clinical characteristics and ZIP code level percent below poverty, there are still issues with residual confounding. Known risk factors, such as maternal alcohol use, illicit drug use, mother’s mobility information during pregnancy, previous preterm birth, and previous preeclampsia information, were unavailable from the Kansas birth certificate data. Fourth, we had about 25% GDM missingness from the birth records data, and the GDM results were based on subjects without GDM missingness. We did not perform any imputation for this missingness because we did not know the mechanism of this missingness. Even though we did not expect the missingness to be correlated with air pollution levels, the GDM results should be interpreted carefully. Last, our results were based on live birth only, and we didn’t consider the effects of air pollutants on pregnancy loss and fetal death. However, air pollution to fetal death is an emerging field, and current studies do not provide consistent results^57–60^.

For future research, it is possible that the true exposure window does not fit neatly within the boundaries of a trimester, and our trimester specific analysis may miss this association. Therefore, future work can explore exposures at finer temporal scale (e.g., months or weeks) to better identify critical exposure window. Also, since the Kansas air pollutant exposure ranges are relatively small, we were unable to assess the non-linearity associations. But we recommend future studies consider this.

## Conclusions

In summary, our findings indicate significant relationships between higher O_3_ exposure during pregnancy and the increased risk of preterm birth and gestational hypertension, as well as decreased birth weight. We also noted that early-pregnancy NO_2_ exposure elevated the risk of GDM. Our study contributes to the body of knowledge regarding O_3_ detrimental impact on public health. Additionally, it demonstrates the significance of reducing exposure to air pollution in pregnant women for the purpose of lower the risk of adverse birth outcomes and pregnancy complications. Personal exposure assessment studies will be required to validate these connections.

### Supplementary Information

Supplementary Information.

## Data Availability

The exposure datasets generated and/or analyzed during the current study are available in the NASA EARTHDATA repository, https://sedac.ciesin.columbia.edu/data/set/aqdh-pm2-5- concentrations-contiguous-us-1-km-2000–2016; https://sedac.ciesin.columbia.edu/data/set/aqdh-o3-concentrations-contiguous-us-1-km2000-2016; https://sedac.ciesin.columbia.edu/data/set/aqdh-no2-concentrationscontiguous-us-1-km-2000-2016
