# Supplementary material for: Effects of air pollution on adverse birth outcomes and pregnancy complications in the U.S. state of Kansas (2000–2015)
Source: Sci Rep. 2023 Dec 6;13:21476. doi: 10.1038/s41598-023-48329-5 (PMC10697947; doi:10.1038/s41598-023-48329-5)

Supplementary Appendix for

Effects of Air Pollution on Adverse Birth Outcomes and Pregnancy Complications in the U.S. State of Kansas (2000-2015)

Hua Hao[1, *], Ph.D., Sodahm R Yoo[2], M.S., Matthew J Strickland[3], Ph.D., Lyndsey A Darrow[3], Ph.D., Rohan R D'Souza[2], M.S., Joshua L Warren[4], Ph.D., Shannon Moss[5], M.P.H., Huaqing Wang[6], Ph.D., Haisu Zhang[1], M.S., Howard H Chang[1, 2], Ph.D.

[1]Gangarosa Department of Environmental Health, Rollins School of Public Health, Emory University, Atlanta, Georgia, 30322, USA

[2]Department of Biostatistics and Bioinformatics, Rollins School of Public Health, Emory University, Atlanta, Georgia, 30322, USA

[3]Depatment of Health Analytics and Biostatistics, Epidemiology and Environmental Health, School of Public Health, University of Nevada, Nevada, Reno, 89557, USA

[4]Department of Biostatistics, School of Medicine, Yale University, New Haven, Connecticut, 06510, USA

[5]Centers for Disease Control and Prevention, Atlanta, Georgia, 30329, USA

[6]Department of Landscape Architecture and Environment Planning, College of Agriculture and Applied Sciences, Utah State University, Logan, Utah, 84322, USA

[*]**Corresponding author:** Hua Hao (hua.hao@emory.edu), 1518 Clifton Rd., NE, Atlanta, GA 30322. Telephone (404) 712-4627

Table S1. Maternal and infant characteristics from birth records of birth weight cohort, gestational diabetes (GDM) cohort, and gestational hypertension (GH) cohort singleton births in Kansas with an estimated date of conception from January 1st, 2000, to December 31st, 2015.

Maternal/Infant Characteristics	Birth Weight Cohort (N=554,787)	GDM Cohort (N=449,871)	GH Cohort (N=590,574)
**Gestational Age (weeks)**			
Mean (SD)	39.1 (1.1)	38.7 (1.6)	38.8 (1.7)
**Maternal Education Level**			
<High School	91,477 (16.6)	72,446 (16.2)	98,927 (16.8)
High School/GED	136,275 (24.7)	105,952 (23.7)	146,482 (24.9)
Some College/AA	159,040 (28.8)	135,801 (30.4)	168,882 (28.7)
BA+	165,156 (29.9)	132,687 (29.7)	173,311 (29.5)
**Maternal Ethnicity**			
Hispanic	137,910 (25.0)	108,061 (24.2)	148,196 (25.2)
Non-Hispanic	416,887 (75.0)		
**Maternal Age (years)**			
Mean (SD)	27.04 (5.8)	27.1 (5.7)	27.0 (5.8)
**Maternal Marital Status**			
Married	268,834 (48.5)	285,053 (63.4)	283,654 (48.0)
Unmarried	152,110 (27.4)	164,437 (36.6)	163,930 (27.8)
Missing	133,843 (24.1)	381 (0.1)	142,990 (24.2)
**Parity**			
1	175,801 (50.7)	140,464 (49.9)	185,692 (50.4)
2	100,916 (29.1)	81,882 (29.1)	107,076 (29.1)
≥3	70,286 (20.3)	59,354 (21.1)	75,809 (20.6)
**Smoking during Pregnancy**			
Yes	48,651 (8.8)	53,336 (11.9)	53,130 (9.0)
No	506,136 (91.2)		
**Conception Season**			
Winter	137,952 (24.9)	108,363 (24.1)	146,831 (24.9)
Spring	136,006 (24.5)	110,000 (24.5)	145,094 (24.6)
Summer	137,215 (24.7)	113,220 (25.2)	146,060 (24.7)
Autumn	143,614 (25.9)	118,288 (26.3)	152,589 (25.8)
**Zip code level percent below poverty**			
Mean (SD)	0.1 (0.1)	0.1 (0.1)	0.1 (0.1)
**Census Tract Level Percentage of Greenspace**			
Mean (SD)	57.7 (33.5)	57.7 (33.5)	57.7 (33.5)

Table S2. Distributions (minimum, maximum, mean and percentiles) of 3 pollutants (NO_2, $PM_{2.5}$, O_3) by different exposure windows with an estimated date of conception from January 1st, 2000, to December 31st, 2015. W4: First 4 weeks. W6: First 6 weeks. T1: First trimester. T2: Second trimester. T3: Third trimester. Total: Total pregnancy.

Pollutants	Minimum	0.5th	1st	25th	50th	75th	99th	99.5th	Maximum	Mean
W4.NO_2	1.45	5.19	6.09	13.50	18.70	24.77	37.86	40.09	70.68	19.49
W6.NO_2	1.47	5.34	6.21	13.59	18.77	24.74	37.19	39.43	63.95	19.49
T1.NO_2	1.71	5.57	6.46	13.74	18.74	24.64	36.36	38.01	56.14	19.48
T2.NO_2	1.73	5.49	6.39	13.60	18.56	24.48	36.32	37.94	57.06	19.35
T3.NO_2	1.58	5.35	6.24	13.37	18.45	24.38	36.52	38.36	56.85	19.20
Total.NO_2	2.21	6.00	6.98	14.07	18.46	24.22	34.38	35.13	42.65	19.35
W4.$PM_{2.5}$	2.00	4.04	4.39	7.82	9.49	11.38	17.17	18.35	23.53	9.70
W6.$PM_{2.5}$	2.47	4.26	4.60	7.97	9.54	11.27	16.25	16.83	19.70	9.69
T1.$PM_{2.5}$	3.11	4.65	4.97	8.22	9.63	11.08	14.80	15.29	17.75	9.67
T2.$PM_{2.5}$	2.54	4.51	4.87	8.14	9.57	11.03	14.76	15.25	17.88	9.60
T3.$PM_{2.5}$	2.29	4.35	4.71	8.01	9.49	11.05	15.15	15.60	23.45	9.56
Total.$PM_{2.5}$	3.17	5.22	5.52	8.36	9.69	10.95	13.41	13.67	15.20	9.62
W4.O_3	11.33	18.43	19.92	31.17	41.13	46.98	59.90	61.29	69.25	39.71
W6.O_3	11.57	18.73	20.35	31.30	41.11	46.86	59.10	60.32	67.01	39.70
T1.O_3	11.96	19.88	21.44	32.38	40.69	46.20	56.82	57.60	62.09	39.71
T2.O_3	12.17	19.99	21.64	32.74	41.05	46.26	56.81	57.59	62.09	39.93
T3.O_3	11.76	19.50	20.96	32.32	41.69	46.58	57.91	58.75	65.43	40.06
Total.O_3	19.32	27.42	29.14	36.84	39.69	43.06	49.35	49.85	55.51	39.89

Table S3. Adjusted ORs or mean differences and 95% CIs for preterm birth, birth weight, GDM and GH per IQR increase in 3 ambient air pollutants in Kansas, U.S.A., with an estimated date of conception from January 1st, 2000, to December 31st, 2015.

Outcome	Pollutant	Period of pregnancy	Adjusted OR/mean difference (95% CI)
Preterm Birth	NO_2	First 4 weeks	1.010 (0.983, 1.037)
		First 6 weeks	1.012 (0.984, 1.041)
		First Trimester	1.010 (0.981, 1.039)
		Second Trimester	0.998 (0.970, 1.027)
		Third Trimester	0.997 (0.970, 1.025)
		Total Pregnancy	1.002 (0.974, 1.032)
	$PM_{2.5}$	First 4 weeks	1.003 (0.981, 1.026)
		First 6 weeks	0.997 (0.974, 1.021)
		First Trimester	0.993 (0.969, 1.017)
		Second Trimester	0.997 (0.973, 1.022)
		Third Trimester	0.984 (0.962, 1.006)
		Total Pregnancy	0.987 (0.958, 1.016)
	O_3	First 4 weeks	0.995 (0.960, 1.031)
		First 6 weeks	1.005 (0.969, 1.043)
		First Trimester	1.022 (0.985, 1.060)
		Second Trimester	1.051 (1.014, 1.089)
		Third Trimester	1.035 (1.002, 1.070)
		Total Pregnancy	1.048 (1.022, 1.075)
Birth weight	NO_2	First 4 weeks	0.994 (-2.185, 4.173)
		First 6 weeks	1.584 (-1.671, 4.840)
		First Trimester	2.884 (-0.475, 6.244)
		Second Trimester	3.371 (0.015, 6.727)
		Third Trimester	1.392 (-2.047, 4.831)
		Total Pregnancy	-2.117 (-4.455, 0.221)
	$PM_{2.5}$	First 4 weeks	0.739 (-1.852, 3.330)
		First 6 weeks	0.593 (-2.117, 3.303)
		First Trimester	2.798 (-0.035, 5.631)
		Second Trimester	-0.189 (-3.071, 2.694)
		Third Trimester	1.962 (-1.091, 5.015)
		Total Pregnancy	1.260 (-1.595, 4.115)
	O_3	First 4 weeks	-1.279 (-5.421, 2.864)
		First 6 weeks	-1.092 (-5.407, 3.222)
		First Trimester	-1.213 (-5.494, 3.067)
		Second Trimester	-9.857 (-14.022, -5.693)
		Third Trimester	-7.934 (-12.340, -3.528)
		Total Pregnancy	-0.671 (-3.343, 2.002)
Gestational Diabetes	NO_2	First 4 weeks	1.044 (0.989, 1.102)

		First 6 weeks	1.044 (0.992, 1.098)
		First Trimester	1.059 (1.002, 1.120)
		Second Trimester	1.014 (0.959, 1.073)
	$PM_{2.5}$	First 4 weeks	1.002 (0.968, 1.036)
		First 6 weeks	0.991 (0.956, 1.027)
		First Trimester	0.990 (0.952, 1.029)
		Second Trimester	0.989 (0.951, 1.029)
	O_3	First 4 weeks	1.034 (0.982, 1.089)
		First 6 weeks	1.014 (0.961, 1.071)
		First Trimester	1.019 (0.964, 1.077)
		Second Trimester	1.041 (0.986, 1.099)
Gestational Hypertension	NO_2	First 4 weeks	0.970 (0.929, 1.013)
		First 6 weeks	0.968 (0.927, 1.012)
		First Trimester	0.967 (0.924, 1.013)
		Second Trimester	0.985 (0.941, 1.031)
		Third Trimester	1.000 (0.954, 1.047)
		Total Pregnancy	0.997 (0.963, 1.031)
	$PM_{2.5}$	First 4 weeks	1.006 (0.972, 1.042)
		First 6 weeks	1.006 (0.970, 1.043)
		First Trimester	1.004 (0.967, 1.044)
		Second Trimester	0.982 (0.945, 1.021)
		Third Trimester	0.961 (0.923, 1.002)
		Total Pregnancy	0.998 (0.956, 1.041)
	O_3	First 4 weeks	1.089 (1.031, 1.149)
		First 6 weeks	1.106 (1.045, 1.170)
		First Trimester	1.107 (1.046, 1.172)
		Second Trimester	1.031 (0.976, 1.089)
		Third Trimester	0.991 (0.936, 1.050)
		Total Pregnancy	1.017 (0.977, 1.057)

Models were adjusted for maternal education level, race, ethnicity, age, marital status, smoking, infant parity, zip code level percent below poverty, census track level greenness, season of conception, and long-term trend using a natural cubic spline on conception date. IQRs: First 4 Weeks NO_2: 11.26 ($\mu g/m^3$); First 6 Weeks NO_2: 11.16 ($\mu g/m^3$); First Trimester NO_2: 10.90 ($\mu g/m^3$); Second Trimester NO_2: 10.88 ($\mu g/m^3$); Third Trimester NO_2: 11.01 ($\mu g/m^3$); Total Pregnancy NO_2: 10.16 ($\mu g/m^3$); First 4 Weeks $PM_{2.5}$: 3.56 (ppb); First 6 Weeks $PM_{2.5}$: 3.30 (ppb); First Trimester $PM_{2.5}$: 2.86 (ppb); Second Trimester $PM_{2.5}$: 2.89 (ppb); Third Trimester $PM_{2.5}$: 3.04 (ppb); Total Pregnancy $PM_{2.5}$: 2.86 (ppb); First 4 Weeks O_3: 15.81 (ppb); First 6 Weeks O_3: 15.56 (ppb); First Trimester O_3: 13.82 (ppb); Second Trimester O_3: 13.51 (ppb); Third Trimester O_3: 14.26 (ppb); Total Pregnancy O_3: 6.23 (ppb).

Table S4. Stratum-specific adjusted ORs or mean difference and 95% CI for preterm birth, birth weight, GDM and GH per interquartile range (IQR) increase in 3 pollutants (NO_2, $PM_{2.5}$, and O_3) in Kansas, U.S.A., with an estimated date of conception from January 1st, 2000, to December 31st, 2015.

Outcome	Pollutant	Stratification	Period of Pregnancy	Stratum	Adjusted OR/mean difference (95% CI)
Preterm	NO_2	Race	First Trimester	White	1.004 (0.973, 1.037)
				Black	1.001 (0.917, 1.092)
				Asian	1.070 (0.828, 1.382)
				Other	1.171 (1.019, 1.344)
			Second Trimester	White	0.999 (0.967, 1.031)
				Black	0.984 (0.901, 1.074)
				Asian	0.986 (0.753, 1.291)
				Other	1.071 (0.929, 1.235)
			Third Trimester	White	0.997 (0.967, 1.028)
				Black	0.99 (0.913, 1.074)
				Asian	0.951 (0.743, 1.219)
				Other	1.069 (0.937, 1.220)
		Hispanic	First Trimester	Hispanic	1.021 (0.969, 1.075)
				Non-Hispanic	1.005 (0.970, 1.040)
			Second Trimester	Hispanic	1.003 (0.952, 1.056)
				Non-Hispanic	0.996 (0.962, 1.031)
			Third Trimester	Hispanic	0.982 (0.934, 1.032)
				Non-Hispanic	1.004 (0.971, 1.038)
		Age	First Trimester	≤27 years	1.011 (0.970, 1.053)
				>27 years	1.004 (0.964, 1.045)
			Second Trimester	≤27 years	1.016 (0.974, 1.058)
				>27 years	0.976 (0.937, 1.016)
			Third Trimester	≤27 years	1.017 (0.977, 1.057)
				>27 years	0.973 (0.936, 1.012)
		Zip Code Level Poverty	First Trimester	≤12%	0.975 (0.935, 1.017)
				>12%	1.042 (1.001, 1.084)
			Second Trimester	≤12%	0.968 (0.929, 1.010)
				>12%	1.026 (0.985, 1.068)
			Third Trimester	≤12%	0.964 (0.926, 1.004)
				>12%	1.029 (0.991, 1.070)
	$PM_{2.5}$	Race	First Trimester	White	1.003 (0.906, 1.111)
				Black	0.757 (0.555, 1.033)
				Asian	0.711 (0.333, 1.519)
				Other	1.127 (0.759, 1.673)
			Second Trimester	White	0.982 (0.886, 1.089)

			Black	1.067 (0.786, 1.449)
			Asian	1.279 (0.598, 2.736)
			Other	1.030 (0.695, 1.526)
		Third Trimester	White	0.973 (0.889, 1.065)
			Black	0.906 (0.702, 1.170)
			Asian	0.687 (0.359, 1.315)
			Other	0.751 (0.533, 1.059)
	Hispanic	First Trimester	Hispanic	0.941 (0.791, 1.118)
			Non-Hispanic	0.979 (0.877, 1.093)
		Second Trimester	Hispanic	0.944 (0.795, 1.122)
			Non-Hispanic	1.001 (0.896, 1.119)
		Third Trimester	Hispanic	0.959 (0.826, 1.115)
			Non-Hispanic	0.935 (0.849, 1.031)
	Age	First Trimester	≤27 years	0.953 (0.834, 1.088)
			>27 years	0.993 (0.872, 1.132)
		Second Trimester	≤27 years	0.999 (0.875, 1.142)
			>27 years	0.972 (0.853, 1.107)
		Third Trimester	≤27 years	0.908 (0.808, 1.021)
			>27 years	0.975 (0.87, 1.092)
	Zip Code Level Poverty	First Trimester	≤12%	0.921 (0.804, 1.055)
			>12%	1.013 (0.890, 1.152)
		Second Trimester	≤12%	0.950 (0.828, 1.089)
			>12%	1.016 (0.893, 1.156)
		Third Trimester	≤12%	0.922 (0.818, 1.039)
			>12%	0.954 (0.853, 1.068)
O_3	Race	First Trimester	White	1.028 (0.996, 1.062)
			Black	0.972 (0.892, 1.060)
			Asian	0.976 (0.791, 1.204)
			Other	0.930 (0.828, 1.045)
		Second Trimester	White	1.039 (1.006, 1.073)
			Black	1.012 (0.929, 1.102)
			Asian	1.097 (0.883, 1.364)
			Other	1.088 (0.971, 1.220)
		Third Trimester	White	1.032 (1.003, 1.062)
			Black	1.003 (0.932, 1.08)
			Asian	1.157 (0.960, 1.394)
			Other	0.963 (0.869, 1.066)
	Hispanic	First Trimester	Hispanic	0.962 (0.912, 1.014)
			Non-Hispanic	1.037 (1.002, 1.074)
		Second Trimester	Hispanic	0.994 (0.943, 1.048)
			Non-Hispanic	1.060 (1.024, 1.096)
		Third Trimester	Hispanic	1.004 (0.959, 1.052)

				Non-Hispanic	1.036 (1.005, 1.068)
		Age	First Trimester	≤27 years	1.018 (0.976, 1.061)
				>27 years	1.019 (0.979, 1.061)
			Second Trimester	≤27 years	1.044 (1.002, 1.088)
				>27 years	1.040 (0.999, 1.082)
			Third Trimester	≤27 years	1.018 (0.982, 1.056)
				>27 years	1.035 (0.998, 1.072)
		Zip Code Level Poverty	First Trimester	≤12%	1.052 (1.008, 1.098)
				>12%	0.989 (0.951, 1.028)
			Second Trimester	≤12%	1.033 (0.990, 1.079)
				>12%	1.048 (1.008, 1.089)
			Third Trimester	≤12%	1.022 (0.984, 1.061)
				>12%	1.031 (0.996, 1.067)
Birth weight	NO_2	Race	First Trimester	White	2.005 (-1.624, 5.634)
				Black	13.338 (0.943, 25.733)
				Asian	-10.126 (-37.585, 17.333)
				Other	2.865 (-14.511, 20.241)
			Second Trimester	White	1.470 (-2.150, 5.091)
				Black	14.797 (2.361, 27.233)
				Asian	11.718 (-16.749, 40.185)
				Other	17.553 (0.072, 35.033)
			Third Trimester	White	0.370 (-3.342, 4.082)
				Black	10.594 (-2.157, 23.345)
				Asian	5.500 (-23.138, 34.137)
				Other	-0.165 (-18.076, 17.746)
		Hispanic	First Trimester	Hispanic	4.544 (-1.949, 11.037)
				Non-Hispanic	2.386 (-1.550, 6.322)
			Second Trimester	Hispanic	5.173 (-1.309, 11.655)
				Non-Hispanic	2.719 (-1.214, 6.652)
			Third Trimester	Hispanic	0.918 (-5.725, 7.561)
				Non-Hispanic	1.710 (-2.321, 5.741)
		Age	First Trimester	≤27 years	3.322 (-1.613, 8.258)
				>27 years	3.249 (-1.340, 7.838)
			Second Trimester	≤27 years	3.755 (-1.181, 8.692)
				>27 years	3.864 (-0.718, 8.447)
			Third Trimester	≤27 years	2.831 (-2.221, 7.883)
				>27 years	1.162 (-3.539, 5.863)
		Zip Code Level Poverty	First Trimester	≤12%	3.252 (-1.362, 7.865)
				>12%	2.192 (-2.841, 7.225)
			Second Trimester	≤12%	2.216 (-2.364, 6.796)
				>12%	4.404 (-0.661, 9.470)

		Third Trimester	≤12%	2.259 (-2.426, 6.945)
			>12%	0.150 (-5.051, 5.351)
$PM_{2.5}$	Race	First Trimester	White	11.030 (-0.637, 22.697)
			Black	-3.175 (-46.219, 39.868)
			Asian	45.746 (-33.17, 124.661)
			Other	15.648 (-32.026, 63.321)
		Second Trimester	White	-2.628 (-14.374, 9.118)
			Black	-1.623 (-44.358, 41.112)
			Asian	-26.224 (-106.06, 53.613)
			Other	35.979 (-11.698, 83.656)
		Third Trimester	White	8.452 (-3.511, 20.415)
			Black	7.587 (-35.888, 51.062)
			Asian	-1.203 (-81.637, 79.232)
			Other	-10.605 (-58.741, 37.53)
	Hispanic	First Trimester	Hispanic	13.962 (-7.376, 35.300)
			Non-Hispanic	9.768 (-2.779, 22.316)
		Second Trimester	Hispanic	8.205 (-13.139, 29.55)
			Non-Hispanic	-3.394 (-16.030, 9.242)
		Third Trimester	Hispanic	1.040 (-20.766, 22.847)
			Non-Hispanic	9.265 (-3.575, 22.105)
	Age	First Trimester	≤27 years	10.945 (-4.854, 26.743)
			>27 years	9.913 (-4.889, 24.714)
		Second Trimester	≤27 years	10.786 (-5.070, 26.642)
			>27 years	-10.267 (-25.17, 4.636)
		Third Trimester	≤27 years	12.856 (-3.303, 29.016)
			>27 years	1.905 (-13.233, 17.043)
	Zip Code Level Poverty	First Trimester	≤12%	10.264 (-4.505, 25.032)
			>12%	12.091 (-3.934, 28.116)
		Second Trimester	≤12%	-5.025 (-19.930, 9.880)
			>12%	5.004 (-11.047, 21.055)
		Third Trimester	≤12%	9.508 (-5.659, 24.675)
			>12%	4.941 (-11.404, 21.286)
O_3	Race	First Trimester	White	-0.975 (-4.665, 2.716)
			Black	-1.766 (-13.992, 10.460)
			Asian	23.821 (1.097, 46.545)
			Other	-3.781 (-17.686, 10.124)
		Second Trimester	White	-7.963 (-11.634, -4.293)
			Black	-9.583 (-21.598, 2.432)
			Asian	-8.559 (-31.286, 14.167)
			Other	-0.498 (-14.312, 13.316)
		Third Trimester	White	-5.909 (-9.628, -2.189)
			Black	-8.161 (-20.332, 4.010)

				Asian	-40.054 (-62.790, -17.318)
				Other	7.873 (-6.168, 21.915)
		Hispanic	First Trimester	Hispanic	-1.339 (-7.925, 5.247)
				Non-Hispanic	-0.554 (-4.489, 3.381)
			Second Trimester	Hispanic	-5.009 (-11.549, 1.531)
				Non-Hispanic	-8.632 (-12.542, -4.721)
			Third Trimester	Hispanic	-2.309 (-8.929, 4.311)
				Non-Hispanic	-7.259 (-11.224, -3.294)
		Age	First Trimester	≤27 years	0.334 (-4.635, 5.304)
				>27 years	-2.110 (-6.704, 2.485)
			Second Trimester	≤27 years	-5.877 (-10.782, -0.971)
				>27 years	-9.679 (-14.277, -5.081)
			Third Trimester	≤27 years	-5.259 (-10.232, -0.286)
				>27 years	-6.778 (-11.441, -2.115)
		Zip Code Level Poverty	First Trimester	≤12%	-1.194 (-5.884, 3.495)
				>12%	-0.012 (-4.895, 4.871)
			Second Trimester	≤12%	-8.966 (-13.626, -4.306)
				>12%	-6.175 (-11.025, -1.326)
			Third Trimester	≤12%	-5.056 (-9.761, -0.350)
				>12%	-6.461 (-11.395, -1.527)
Gestational Diabetes	NO_2	Race	First Trimester	White	1.020 (0.959, 1.086)
				Black	1.271 (1.027, 1.573)
				Asian	1.155 (0.901, 1.481)
				Other	1.130 (0.948, 1.346)
			Second Trimester	White	0.975 (0.916, 1.039)
				Black	1.114 (0.885, 1.402)
				Asian	1.057 (0.813, 1.375)
				Other	1.202 (1.004, 1.439)
		Hispanic	First Trimester	Hispanic	1.157 (1.051, 1.273)
				Non-Hispanic	1.003 (0.939, 1.072)
			Second Trimester	Hispanic	1.094 (0.990, 1.209)
				Non-Hispanic	0.975 (0.911, 1.043)
		Age	First Trimester	≤27 years	1.085 (0.976, 1.205)
				>27 years	1.049 (0.984, 1.118)
			Second Trimester	≤27 years	0.967 (0.867, 1.078)
				>27 years	1.033 (0.969, 1.103)
		Zip Code Level Poverty	First Trimester	≤12%	1.032 (0.952, 1.119)
				>12%	1.038 (0.962, 1.12)
			Second Trimester	≤12%	0.990 (0.913, 1.074)
				>12%	0.999 (0.923, 1.082)

$PM_{2.5}$	Race	First Trimester	White	0.980 (0.831, 1.155)
			Black	1.235 (0.641, 2.379)
			Asian	0.941 (0.473, 1.872)
			Other	0.721 (0.450, 1.156)
		Second Trimester	White	0.994 (0.842, 1.174)
			Black	0.689 (0.353, 1.344)
			Asian	0.760 (0.375, 1.54)
			Other	0.965 (0.606, 1.537)
	Hispanic	First Trimester	Hispanic	1.215 (0.922, 1.603)
			Non-Hispanic	0.875 (0.735, 1.041)
		Second Trimester	Hispanic	1.272 (0.964, 1.678)
			Non-Hispanic	0.862 (0.723, 1.028)
	Age	First Trimester	≤27 years	0.988 (0.748, 1.305)
			>27 years	0.958 (0.806, 1.138)
		Second Trimester	≤27 years	1.097 (0.830, 1.449)
			>27 years	0.925 (0.777, 1.101)
	Zip Code Level Poverty	First Trimester	≤12%	0.954 (0.773, 1.178)
			>12%	0.973 (0.791, 1.195)
		Second Trimester	≤12%	0.899 (0.725, 1.113)
			>12%	1.044 (0.848, 1.284)
O_3	Race	First Trimester	White	1.010 (0.961, 1.061)
			Black	1.119 (0.938, 1.335)
			Asian	1.124 (0.919, 1.374)
			Other	0.943 (0.822, 1.083)
		Second Trimester	White	1.036 (0.986, 1.089)
			Black	1.029 (0.862, 1.229)
			Asian	0.910 (0.744, 1.114)
			Other	1.071 (0.934, 1.228)
	Hispanic	First Trimester	Hispanic	0.964 (0.891, 1.043)
			Non-Hispanic	1.042 (0.989, 1.098)
		Second Trimester	Hispanic	1.048 (0.968, 1.134)
			Non-Hispanic	1.030 (0.977, 1.086)
	Age	First Trimester	≤27 years	1.071 (0.986, 1.164)
			>27 years	0.993 (0.944, 1.045)
		Second Trimester	≤27 years	1.026 (0.945, 1.114)
			>27 years	1.034 (0.983, 1.089)
	Zip Code Level Poverty	First Trimester	≤12%	1.008 (0.944, 1.076)
			>12%	1.013 (0.955, 1.074)
		Second Trimester	≤12%	1.064 (0.996, 1.136)
			>12%	1.004 (0.947, 1.064)

Gestational Hypertension	NO_2	Race	First Trimester	White	0.974 (0.927, 1.023)
				Black	0.822 (0.699, 0.967)
				Asian	0.983 (0.587, 1.645)
				Other	1.166 (0.926, 1.469)
			Second Trimester	White	0.989 (0.941, 1.039)
				Black	0.898 (0.766, 1.053)
				Asian	1.270 (0.768, 2.099)
				Other	1.016 (0.794, 1.298)
		Hispanic	First Trimester	Hispanic	1.026 (0.937, 1.123)
				Non-Hispanic	0.950 (0.900, 1.002)
			Second Trimester	Hispanic	1.006 (0.919, 1.103)
				Non-Hispanic	0.978 (0.927, 1.031)
		Age	First Trimester	≤27 years	0.958 (0.891, 1.031)
				>27 years	0.975 (0.919, 1.034)
			Second Trimester	≤27 years	0.992 (0.922, 1.067)
				>27 years	0.984 (0.928, 1.044)
		Zip Code Level Poverty	First Trimester	≤12%	0.907 (0.849, 0.968)
				>12%	1.028 (0.964, 1.098)
			Second Trimester	≤12%	0.941 (0.881, 1.005)
				>12%	1.031 (0.965, 1.101)
	$PM_{2.5}$	Race	First Trimester	White	1.013 (0.866, 1.185)
				Black	1.230 (0.707, 2.139)
				Asian	0.809 (0.188, 3.478)
				Other	1.234 (0.611, 2.492)
			Second Trimester	White	0.960 (0.820, 1.124)
				Black	0.944 (0.547, 1.627)
				Asian	0.460 (0.104, 2.040)
				Other	0.833 (0.413, 1.679)
		Hispanic	First Trimester	Hispanic	1.183 (0.876, 1.597)
				Non-Hispanic	0.979 (0.827, 1.158)
			Second Trimester	Hispanic	1.029 (0.763, 1.388)
				Non-Hispanic	0.911 (0.769, 1.078)
		Age	First Trimester	≤27 years	1.008 (0.797, 1.273)
				>27 years	1.026 (0.850, 1.238)
			Second Trimester	≤27 years	0.987 (0.782, 1.247)
				>27 years	0.909 (0.753, 1.098)
		Zip Code Level Poverty	First Trimester	≤12%	0.842 (0.684, 1.038)
				>12%	1.237 (1.005, 1.522)
			Second Trimester	≤12%	0.745 (0.604, 0.918)
				>12%	1.190 (0.967, 1.465)

O_3	Race	First Trimester	White	1.085 (1.033, 1.138)
			Black	1.073 (0.921, 1.250)
			Asian	1.564 (1.047, 2.337)
			Other	0.968 (0.789, 1.188)
		Second Trimester	White	1.042 (0.993, 1.093)
			Black	1.023 (0.882, 1.186)
			Asian	0.667 (0.440, 1.012)
			Other	0.881 (0.721, 1.077)
	Hispanic	First Trimester	Hispanic	1.118 (1.022, 1.222)
			Non-Hispanic	1.071 (1.017, 1.128)
		Second Trimester	Hispanic	1.028 (0.941, 1.122)
			Non-Hispanic	1.024 (0.973, 1.078)
	Age	First Trimester	≤27 years	1.081 (1.005, 1.161)
			>27 years	1.086 (1.026, 1.149)
		Second Trimester	≤27 years	1.004 (0.935, 1.077)
			>27 years	1.044 (0.986, 1.105)
	Zip Code Level Poverty	First Trimester	≤12%	1.072 (1.004, 1.144)
			>12%	1.095 (1.030, 1.165)
		Second Trimester	≤12%	1.059 (0.993, 1.130)
			>12%	0.996 (0.937, 1.058)

Models were adjusted the same covariates as the main model besides the stratification variable. IQRs: First 4 Weeks NO_2: 11.26 (μg/m^3); First 6 Weeks NO_2: 11.16 (μg/m^3); First Trimester NO_2: 10.90 (μg/m^3); Second Trimester NO_2: 10.88 (μg/m^3); Third Trimester NO_2: 11.01 (μg/m^3); Total Pregnancy NO_2: 10.16 (μg/m^3); First 4 Weeks $PM_{2.5}$: 3.56 (ppb); First 6 Weeks $PM_{2.5}$: 3.30 (ppb); First Trimester $PM_{2.5}$: 2.86 (ppb); Second Trimester $PM_{2.5}$: 2.89 (ppb); Third Trimester $PM_{2.5}$: 3.04 (ppb); Total Pregnancy $PM_{2.5}$: 2.86 (ppb); First 4 Weeks O_3: 15.81 (ppb); First 6 Weeks O_3: 15.56 (ppb); First Trimester O_3: 13.82 (ppb); Second Trimester O_3: 13.51 (ppb); Third Trimester O_3: 14.26 (ppb); Total Pregnancy O_3: 6.23 (ppb).

Table S5. Sensitivity analysis of adjusted ORs or mean differences and 95% CIs for preterm birth, birth weight, GDM and GH per IQR increase in 3 ambient air pollutants in Kansas, U.S.A., with an estimated date of conception from January 1st, 2000, to December 31st, 2015 for different amounts of smoothing on conception date (degree of freedom = 5, 8, 10 or adjusted conception year as a character variable).

Outcome	Pollutant	Period of pregnancy	Adjusted OR/mean difference (95% CI) DF=5	Adjusted OR/mean difference (95% CI) DF=8	Adjusted OR/mean difference (95% CI) DF=10	Adjusted OR/mean difference (95% CI) Conception year as a factor variable
Preterm Birth	NO_2	First 4 weeks	1.010 (0.983, 1.037)	1.010 (0.983, 1.037)	1.003 (0.976, 1.031)	1.005 (0.978, 1.033)
		First 6 weeks	1.012 (0.984, 1.041)	1.012 (0.984, 1.041)	1.005 (0.977, 1.034)	1.007 (0.979, 1.036)
		First Trimester	1.010 (0.981, 1.039)	1.010 (0.981, 1.039)	1.002 (0.974, 1.032)	1.005 (0.976, 1.034)
		Second Trimester	0.998 (0.970, 1.027)	0.998 (0.970, 1.027)	0.991 (0.963, 1.020)	0.994 (0.965, 1.023)
		Third Trimester	0.997 (0.970, 1.025)	0.992 (0.965, 1.020)	0.990 (0.963, 1.018)	0.991 (0.963, 1.019)
		Total Pregnancy	1.002 (0.974, 1.032)	0.996 (0.968, 1.025)	0.995 (0.966, 1.024)	0.997 (0.968, 1.026)
	$PM_{2.5}$	First 4 weeks	1.003 (0.981, 1.026)	1.003 (0.981, 1.026)	0.998 (0.976, 1.021)	0.996 (0.973, 1.019)
		First 6 weeks	0.997 (0.974, 1.021)	0.997 (0.974, 1.021)	0.992 (0.969, 1.016)	0.990 (0.967, 1.014)
		First Trimester	0.993 (0.969, 1.017)	0.993 (0.969, 1.017)	0.990 (0.966, 1.015)	0.993 (0.968, 1.018)
		Second Trimester	0.997 (0.973, 1.022)	0.997 (0.972, 1.022)	1.004 (0.979, 1.029)	1.001 (0.975, 1.027)
		Third Trimester	0.984 (0.962, 1.006)	0.987 (0.965, 1.009)	0.993 (0.971, 1.016)	0.986 (0.963, 1.009)
		Total Pregnancy	0.987 (0.958, 1.016)	0.991 (0.962, 1.020)	0.994 (0.965, 1.024)	0.990 (0.960, 1.021)
	O_3	First 4 weeks	0.995 (0.960, 1.031)	0.995 (0.960, 1.031)	0.986 (0.951, 1.022)	0.986 (0.951, 1.022)
		First 6 weeks	1.005 (0.969, 1.043)	1.005 (0.968, 1.043)	0.994 (0.958, 1.032)	0.993 (0.956, 1.031)
		First Trimester	1.022 (0.985, 1.060)	1.022 (0.985, 1.060)	1.009 (0.972, 1.048)	1.006 (0.969, 1.045)
		Second Trimester	1.051 (1.014, 1.089)	1.052 (1.015, 1.090)	1.035 (0.998, 1.074)	1.041 (1.003, 1.080)
		Third Trimester	1.035 (1.002, 1.070)	1.025 (0.992, 1.060)	1.017 (0.983, 1.051)	1.024 (0.991, 1.059)
		Total Pregnancy	1.048 (1.022, 1.075)	1.040 (1.012, 1.069)	1.032 (1.004, 1.060)	1.037 (1.008, 1.066)
Birth weight	NO_2	First 4 weeks	0.994 (-2.185, 4.173)	1.432 (-1.758, 4.621)	1.519 (-1.671, 4.708)	1.751 (-1.443, 4.944)
		First 6 weeks	1.584 (-1.671, 4.840)	2.061 (-1.207, 5.329)	2.162 (-1.106, 5.429)	2.421 (-0.853, 5.695)
		First Trimester	2.884 (-0.475, 6.244)	3.415 (0.040, 6.789)	3.518 (0.143, 6.892)	3.713 (0.329, 7.096)
		Second Trimester	3.371 (0.015, 6.727)	3.910 (0.540, 7.279)	4.012 (0.640, 7.384)	3.899 (0.507, 7.290)

		Third Trimester	1.392 (-2.047, 4.831)	1.969 (-1.485, 5.424)	2.083 (-1.375, 5.541)	1.730 (-1.739, 5.199)
		Total Pregnancy	-2.117 (-4.455, 0.221)	-2.094 (-4.432, 0.244)	-2.101 (-4.439, 0.237)	-2.096 (-4.434, 0.242)
	$PM_{2.5}$	First 4 weeks	0.739 (-1.852, 3.330)	0.819 (-1.777, 3.415)	1.071 (-1.541, 3.684)	1.681 (-0.981, 4.344)
		First 6 weeks	0.593 (-2.117, 3.303)	0.676 (-2.039, 3.390)	0.968 (-1.768, 3.703)	1.532 (-1.257, 4.321)
		First Trimester	2.798 (-0.035, 5.631)	2.747 (-0.089, 5.583)	3.099 (0.238, 5.959)	3.310 (0.381, 6.240)
		Second Trimester	-0.189 (-3.071, 2.694)	-0.387 (-3.281, 2.506)	-0.383 (-3.296, 2.531)	-0.488 (-3.478, 2.502)
		Third Trimester	1.962 (-1.091, 5.015)	1.749 (-1.313, 4.811)	1.433 (-1.671, 4.537)	1.374 (-1.844, 4.591)
		Total Pregnancy	1.260 (-1.595, 4.115)	1.259 (-1.596, 4.114)	1.262 (-1.593, 4.118)	1.274 (-1.581, 4.130)
	O_3	First 4 weeks	-1.279 (-5.421, 2.864)	-0.200 (-4.404, 4.003)	-0.279 (-4.483, 3.925)	0.200 (-4.025, 4.426)
		First 6 weeks	-1.092 (-5.407, 3.222)	0.179 (-4.209, 4.566)	0.124 (-4.263, 4.512)	0.827 (-3.594, 5.248)
		First Trimester	-1.213 (-5.494, 3.067)	0.316 (-4.062, 4.695)	0.278 (-4.099, 4.655)	1.410 (-3.036, 5.856)
		Second Trimester	-9.857 (-14.022, -5.693)	-8.736 (-13.008, -4.464)	-8.648 (-12.92, -4.376)	-8.422 (-12.736, -4.108)
		Third Trimester	-7.934 (-12.34, -3.528)	-6.781 (-11.279, -2.283)	-6.387 (-10.91, -1.863)	-7.439 (-11.972, -2.906)
		Total Pregnancy	-0.671 (-3.343, 2.002)	-0.682 (-3.354, 1.990)	-0.682 (-3.354, 1.990)	-0.687 (-3.360, 1.985)
Gestational Diabetes	NO_2	First 4 weeks	1.044 (0.989, 1.102)	1.044 (0.989, 1.103)	1.047 (0.992, 1.106)	1.041 (0.985, 1.099)
		First 6 weeks	1.044 (0.992, 1.098)	1.045 (0.993, 1.100)	1.048 (0.996, 1.103)	1.041 (0.990, 1.096)
		First Trimester	1.059 (1.002, 1.120)	1.061 (1.003, 1.122)	1.064 (1.006, 1.126)	1.057 (1.000, 1.118)
		Second Trimester	1.014 (0.959, 1.073)	1.016 (0.960, 1.074)	1.017 (0.961, 1.076)	1.013 (0.957, 1.072)
	$PM_{2.5}$	First 4 weeks	1.002 (0.968, 1.036)	1.003 (0.97, 1.038)	1.012 (0.977, 1.048)	1.004 (0.970, 1.040)
		First 6 weeks	0.991 (0.956, 1.027)	0.993 (0.958, 1.029)	1.003 (0.966, 1.041)	0.995 (0.959, 1.032)
		First Trimester	0.990 (0.952, 1.029)	0.992 (0.954, 1.031)	1.006 (0.966, 1.049)	0.999 (0.960, 1.039)
		Second Trimester	0.989 (0.951, 1.029)	0.991 (0.952, 1.031)	1.005 (0.964, 1.047)	0.996 (0.956, 1.036)
	O_3	First 4 weeks	1.034 (0.982, 1.089)	1.034 (0.982, 1.090)	1.027 (0.975, 1.083)	1.031 (0.978, 1.086)
		First 6 weeks	1.014 (0.961, 1.071)	1.015 (0.961, 1.072)	1.008 (0.954, 1.065)	1.010 (0.956, 1.067)
		First Trimester	1.019 (0.964, 1.077)	1.021 (0.965, 1.079)	1.013 (0.958, 1.072)	1.016 (0.960, 1.075)
		Second Trimester	1.041 (0.986, 1.099)	1.044 (0.988, 1.104)	1.042 (0.986, 1.101)	1.051 (0.995, 1.111)
Gestational Hypertension	NO_2	First 4 weeks	0.970 (0.929, 1.013)	0.967 (0.926, 1.010)	0.967 (0.926, 1.009)	0.967 (0.926, 1.009)
		First 6 weeks	0.968 (0.927, 1.012)	0.965 (0.923, 1.009)	0.964 (0.923, 1.008)	0.964 (0.923, 1.008)

	First Trimester	0.967 (0.924, 1.013)	0.963 (0.920, 1.008)	0.962 (0.919, 1.007)	0.962 (0.919, 1.007)
	Second Trimester	0.985 (0.941, 1.031)	0.981 (0.936, 1.027)	0.978 (0.934, 1.024)	0.978 (0.934, 1.024)
	Third Trimester	1.000 (0.954, 1.047)	0.994 (0.949, 1.042)	0.991 (0.946, 1.039)	0.991 (0.946, 1.039)
	Total Pregnancy	0.997 (0.963, 1.031)	0.997 (0.963, 1.031)	0.997 (0.963, 1.031)	0.997 (0.963, 1.031)
$PM_{2.5}$	First 4 weeks	1.006 (0.972, 1.042)	1.008 (0.974, 1.044)	1.003 (0.968, 1.039)	1.003 (0.968, 1.039)
	First 6 weeks	1.006 (0.970, 1.043)	1.008 (0.972, 1.046)	1.002 (0.966, 1.040)	1.002 (0.966, 1.040)
	First Trimester	1.004 (0.967, 1.044)	1.008 (0.970, 1.047)	1.000 (0.962, 1.040)	1.000 (0.962, 1.040)
	Second Trimester	0.982 (0.945, 1.021)	0.987 (0.949, 1.026)	0.980 (0.942, 1.020)	0.980 (0.942, 1.020)
	Third Trimester	0.961 (0.923, 1.002)	0.961 (0.923, 1.002)	0.961 (0.922, 1.002)	0.961 (0.922, 1.002)
	Total Pregnancy	0.998 (0.956, 1.041)	0.998 (0.956, 1.041)	0.998 (0.956, 1.041)	0.998 (0.956, 1.041)
O_3	First 4 weeks	1.089 (1.031, 1.149)	1.080 (1.022, 1.141)	1.084 (1.026, 1.145)	1.084 (1.026, 1.145)
	First 6 weeks	1.106 (1.045, 1.170)	1.097 (1.036, 1.162)	1.101 (1.040, 1.166)	1.101 (1.040, 1.166)
	First Trimester	1.107 (1.046, 1.172)	1.098 (1.036, 1.163)	1.102 (1.040, 1.168)	1.102 (1.040, 1.168)
	Second Trimester	1.031 (0.976, 1.089)	1.018 (0.962, 1.077)	1.021 (0.965, 1.081)	1.021 (0.965, 1.081)
	Third Trimester	0.991 (0.936, 1.050)	0.978 (0.922, 1.038)	0.978 (0.922, 1.038)	0.978 (0.922, 1.038)
	Total Pregnancy	1.017 (0.977, 1.057)	1.016 (0.977, 1.057)	1.016 (0.977, 1.057)	1.016 (0.977, 1.057)

Models were adjusted for maternal education level, race, ethnicity, age, marital status, smoking, infant parity, zip code level percent below poverty, census track level greenness, season of conception, and long-term trend using a natural cubic spline on conception date. IQRs: First 4 Weeks NO_2: 11.26 (μg/m^3); First 6 Weeks NO_2: 11.16 (μg/m^3); First Trimester NO_2: 10.90 (μg/m^3); Second Trimester NO_2: 10.88 (μg/m^3); Third Trimester NO_2: 11.01 (μg/m^3); Total Pregnancy NO_2: 10.16 (μg/m^3); First 4 Weeks $PM_{2.5}$: 3.56 (ppb); First 6 Weeks $PM_{2.5}$: 3.30 (ppb); First Trimester $PM_{2.5}$: 2.86 (ppb); Second Trimester $PM_{2.5}$: 2.89 (ppb); Third Trimester $PM_{2.5}$: 3.04 (ppb); Total Pregnancy $PM_{2.5}$: 2.86 (ppb); First 4 Weeks O_3: 15.81 (ppb); First 6 Weeks O_3: 15.56 (ppb); First Trimester O_3: 13.82 (ppb); Second Trimester O_3: 13.51 (ppb); Third Trimester O_3: 14.26 (ppb); Total Pregnancy O_3: 6.23 (ppb).

Table S6. Sensitivity analysis of adjusted ORs or mean differences and 95% CIs for preterm birth after excluded induction labor births per IQR increase in 3 ambient air pollutants in Kansas, U.S.A., with an estimated date of conception from January 1st, 2000, to December 31st, 2015 for different amounts of smoothing on conception date (degree of freedom = 5, 8, 10 or adjusted conception year as a categorical variable).

Outcome	Pollutant	Period of pregnancy	Adjusted OR/mean difference (95% CI) DF=5	Adjusted OR/mean difference (95% CI) DF=8	Adjusted OR/mean difference (95% CI) DF=10	Adjusted OR/mean difference (95% CI) Conception year as a factor variable
Preterm Birth	NO_2	First 4 weeks	1.009 (0.980, 1.039)	1.006 (0.977, 1.036)	1.007 (0.978, 1.036)	1.006 (0.978, 1.036)
		First 6 weeks	1.012 (0.982, 1.042)	1.008 (0.978, 1.039)	1.009 (0.979, 1.039)	1.008 (0.979, 1.039)
		First Trimester	1.010 (0.979, 1.041)	1.006 (0.975, 1.037)	1.007 (0.977, 1.039)	1.007 (0.977, 1.039)
		Second Trimester	1.000 (0.970, 1.031)	0.996 (0.965, 1.027)	0.997 (0.967, 1.029)	0.997 (0.967, 1.029)
		Third Trimester	0.995 (0.966, 1.024)	0.990 (0.962, 1.020)	0.991 (0.963, 1.021)	0.990 (0.961, 1.019)
		Total Pregnancy	1.003 (0.973, 1.034)	0.998 (0.968, 1.029)	1.000 (0.970, 1.031)	0.999 (0.969, 1.031)
	$PM_{2.5}$	First 4 weeks	0.997 (0.973, 1.021)	0.996 (0.972, 1.020)	0.994 (0.971, 1.018)	0.990 (0.966, 1.015)
		First 6 weeks	0.993 (0.969, 1.018)	0.992 (0.968, 1.018)	0.991 (0.966, 1.016)	0.988 (0.963, 1.013)
		First Trimester	0.986 (0.960, 1.012)	0.987 (0.962, 1.013)	0.986 (0.960, 1.012)	0.987 (0.961, 1.014)
		Second Trimester	1.000 (0.974, 1.026)	1.004 (0.977, 1.030)	1.007 (0.981, 1.035)	1.005 (0.977, 1.032)
		Third Trimester	0.985 (0.962, 1.009)	0.988 (0.965, 1.012)	0.993 (0.969, 1.017)	0.988 (0.964, 1.013)
		Total Pregnancy	0.985 (0.955, 1.016)	0.989 (0.959, 1.02)	0.993 (0.962, 1.025)	0.990 (0.958, 1.022)
	O_3	First 4 weeks	0.991 (0.954, 1.029)	0.984 (0.947, 1.023)	0.987 (0.950, 1.026)	0.983 (0.945, 1.022)
		First 6 weeks	1.003 (0.964, 1.044)	0.996 (0.957, 1.037)	0.999 (0.959, 1.040)	0.992 (0.952, 1.033)
		First Trimester	1.023 (0.984, 1.064)	1.015 (0.975, 1.056)	1.018 (0.978, 1.060)	1.007 (0.967, 1.049)
		Second Trimester	1.064 (1.024, 1.105)	1.055 (1.015, 1.097)	1.058 (1.018, 1.100)	1.054 (1.013, 1.097)
		Third Trimester	1.042 (1.006, 1.079)	1.033 (0.997, 1.071)	1.031 (0.995, 1.069)	1.033 (0.997, 1.071)
		Total Pregnancy	1.057 (1.028, 1.086)	1.050 (1.020, 1.082)	1.053 (1.022, 1.084)	1.047 (1.016, 1.078)

Models were adjusted for maternal education level, race, ethnicity, age, marital status, smoking, infant parity, zip code level percent below poverty, census track level greenness, season of conception, and long-term trend using a natural cubic spline on conception date. IQRs: First 4 Weeks NO_2: 11.26 (μg/m^3); First 6 Weeks NO_2: 11.16 (μg/m^3); First Trimester NO_2: 10.90 (μg/m^3); Second Trimester NO_2: 10.88 (μg/m^3); Third Trimester NO_2: 11.01 (μg/m^3); Total Pregnancy NO_2: 10.16 (μg/m^3); First 4 Weeks $PM_{2.5}$: 3.56 (ppb); First 6 Weeks $PM_{2.5}$: 3.30 (ppb); First

Trimester $PM_{2.5}$: 2.86 (ppb); Second Trimester $PM_{2.5}$: 2.89 (ppb); Third Trimester $PM_{2.5}$: 3.04 (ppb); Total Pregnancy $PM_{2.5}$: 2.86 (ppb); First 4 Weeks O_3: 15.81 (ppb); First 6 Weeks O_3: 15.56 (ppb); First Trimester O_3: 13.82 (ppb); Second Trimester O_3: 13.51 (ppb); Third Trimester O_3: 14.26 (ppb); Total Pregnancy O_3: 6.23 (ppb).

Table S7. Sensitivity analysis of adjusted ORs or mean differences and 95% CIs for preterm birth, birth weight, gestational diabetes (GDM) and gestational hypertension (GH) per IQR increase in 3 ambient air pollutants in Kansas, U.S.A., with an estimated date of conception from January 1st, 2000, to December 31st, 2015, by adjusting trimester-average exposures (trimester 1, trimester 2, and trimester 3) simultaneously in the model.

Outcome	Pollutant	Period of pregnancy	Adjusted OR/mean difference (95% CI)
Preterm Birth	NO_2	First Trimester	1.011 (0.974, 1.050)
		Second Trimester	0.981 (0.904, 1.065)
		Third Trimester	1.026 (0.998, 1.055)
	$PM_{2.5}$	First Trimester	0.999 (0.986, 1.012)
		Second Trimester	1.005 (0.983, 1.028)
		Third Trimester	1.003 (0.995, 1.010)
	O_3	First Trimester	1.007 (0.960, 1.057)
		Second Trimester	0.964 (0.880, 1.056)
		Third Trimester	1.041 (1.008, 1.076)
Birthweight	NO_2	First Trimester	0.988 (-4.3, 6.277)
		Second Trimester	5.089 (-1.373, 11.551)
		Third Trimester	-3.282 (-8.732, 2.168)
	$PM_{2.5}$	First Trimester	2.782 (-0.243, 5.808)
		Second Trimester	-1.392 (-4.471, 1.687)
		Third Trimester	1.535 (-1.726, 4.796)
	O_3	First Trimester	-3.594 (-8.513, 1.324)
		Second Trimester	-8.239 (-12.566, -3.911)
		Third Trimester	-8.359 (-13.417, -3.301)
Gestational Diabetes	NO_2	First Trimester	1.067 (1.002, 1.136)
		Second Trimester	0.984 (0.924, 1.049)
	$PM_{2.5}$	First Trimester	0.991 (0.953, 1.030)
		Second Trimester	0.991 (0.952, 1.031)
	O_3	First Trimester	1.016 (0.961, 1.074)

		Second Trimester	1.040 (0.985, 1.098)
Gestational Hypertension	NO_2	First Trimester	0.955 (0.894, 1.020)
		Second Trimester	1.001 (0.924, 1.085)
		Third Trimester	1.024 (0.957, 1.096)
	$PM_{2.5}$	First Trimester	1.019 (0.978, 1.060)
		Second Trimester	0.990 (0.950, 1.032)
		Third Trimester	0.948 (0.908, 0.989)
	O_3	First Trimester	1.125 (1.055, 1.199)
		Second Trimester	1.011 (0.955, 1.070)
		Third Trimester	1.042 (0.976, 1.112)

Models were adjusted for maternal education level, race, ethnicity, age, marital status, smoking, infant parity, zip code level percent below poverty, census track level greenness, season of conception, and long-term trend using a natural cubic spline on conception date. IQRs: First 4 Weeks NO_2: 11.26 (μg/m^3); First 6 Weeks NO_2: 11.16 (μg/m^3); First Trimester NO_2: 10.90 (μg/m^3); Second Trimester NO_2: 10.88 (μg/m^3); Third Trimester NO_2: 11.01 (μg/m^3); Total Pregnancy NO_2: 10.16 (μg/m^3); First 4 Weeks $PM_{2.5}$: 3.56 (ppb); First 6 Weeks $PM_{2.5}$: 3.30 (ppb); First Trimester $PM_{2.5}$: 2.86 (ppb); Second Trimester $PM_{2.5}$: 2.89 (ppb); Third Trimester $PM_{2.5}$: 3.04 (ppb); Total Pregnancy $PM_{2.5}$: 2.86 (ppb); First 4 Weeks O_3: 15.81 (ppb); First 6 Weeks O_3: 15.56 (ppb); First Trimester O_3: 13.82 (ppb); Second Trimester O_3: 13.51 (ppb); Third Trimester O_3: 14.26 (ppb); Total Pregnancy O_3: 6.23 (ppb).

Table S8. Sensitivity analysis of adjusted ORs or mean differences and 95% CIs for preterm birth, birth weight, gestational diabetes (GDM) and gestational hypertension (GH) per IQR increase in 3 ambient air pollutants in Kansas, U.S.A., with an estimated date of conception from January 1st, 2000, to December 31st, 2015, by adjusting three pollutants (NO_2, $PM_{2.5}$, O_3) simultaneously in the model.

Outcome	Period of pregnancy	Pollutant	Adjusted OR/mean difference (95% CI)
Preterm Birth	First Trimester	NO_2	1.016 (0.991, 1.041)
		$PM_{2.5}$	0.991 (0.971, 1.012)
		O_3	1.021 (0.990, 1.053)
	Second Trimester	NO_2	1.012 (0.988, 1.038)
		$PM_{2.5}$	0.998 (0.977, 1.019)
		O_3	1.031 (1.000, 1.062)
	Third Trimester	NO_2	1.014 (0.990, 1.039)
		$PM_{2.5}$	0.989 (0.970, 1.009)
		O_3	1.035 (1.006, 1.065)
Birthweight	First Trimester	NO_2	2.236 (-1.223, 5.694)
		$PM_{2.5}$	2.504 (-0.388, 5.395)
		O_3	-1.036 (-5.380, 3.309)
	Second Trimester	NO_2	2.428 (-1.016, 5.873)
		$PM_{2.5}$	-0.228 (-3.166, 2.709)
		O_3	-9.449 (-13.661, -5.236)
	Third Trimester	NO_2	0.119 (-3.410, 3.648)
		$PM_{2.5}$	2.207 (-0.901, 5.316)
		O_3	-8.064 (-12.521, -3.607)
Gestational Diabetes	First Trimester	NO_2	1.065 (1.007, 1.125)
		$PM_{2.5}$	0.985 (0.947, 1.024)
		O_3	1.033 (0.976, 1.092)

	Second Trimester	NO_2	1.023 (0.967, 1.082)
		$PM_{2.5}$	0.985 (0.947, 1.025)
		O_3	1.047 (0.991, 1.107)
Gestational Hypertension	First Trimester	NO_2	0.978 (0.933, 1.025)
		$PM_{2.5}$	1.002 (0.964, 1.042)
		O_3	1.102 (1.041, 1.168)
	Second Trimester	NO_2	0.992 (0.946, 1.039)
		$PM_{2.5}$	0.982 (0.944, 1.021)
		O_3	1.031 (0.975, 1.090)
	Third Trimester	NO_2	1.008 (0.961, 1.057)
		$PM_{2.5}$	0.949 (0.910, 0.989)
		O_3	0.996 (0.940, 1.056)

Models were adjusted for maternal education level, race, ethnicity, age, marital status, smoking, infant parity, zip code level percent below poverty, census track level greenness, season of conception, and long-term trend using a natural cubic spline on conception date. IQRs: First 4 Weeks NO_2: 11.26 (µg/m^3); First 6 Weeks NO_2: 11.16 (µg/m^3); First Trimester NO_2: 10.90 (µg/m^3); Second Trimester NO_2: 10.88 (µg/m^3); Third Trimester NO_2: 11.01 (µg/m^3); Total Pregnancy NO_2: 10.16 (µg/m^3); First 4 Weeks $PM_{2.5}$: 3.56 (ppb); First 6 Weeks $PM_{2.5}$: 3.30 (ppb); First Trimester $PM_{2.5}$: 2.86 (ppb); Second Trimester $PM_{2.5}$: 2.89 (ppb); Third Trimester $PM_{2.5}$: 3.04 (ppb); Total Pregnancy $PM_{2.5}$: 2.86 (ppb); First 4 Weeks O_3: 15.81 (ppb); First 6 Weeks O_3: 15.56 (ppb); First Trimester O_3: 13.82 (ppb); Second Trimester O_3: 13.51 (ppb); Third Trimester O_3: 14.26 (ppb); Total Pregnancy O_3: 6.23 (ppb).

Figure S1. The correlation matrix of 3 pollutants for preterm birth cohort (N=596,926), Kansas, U.S.A., with an estimated date of conception from January 1st, 2000, to December 31st, 2015.

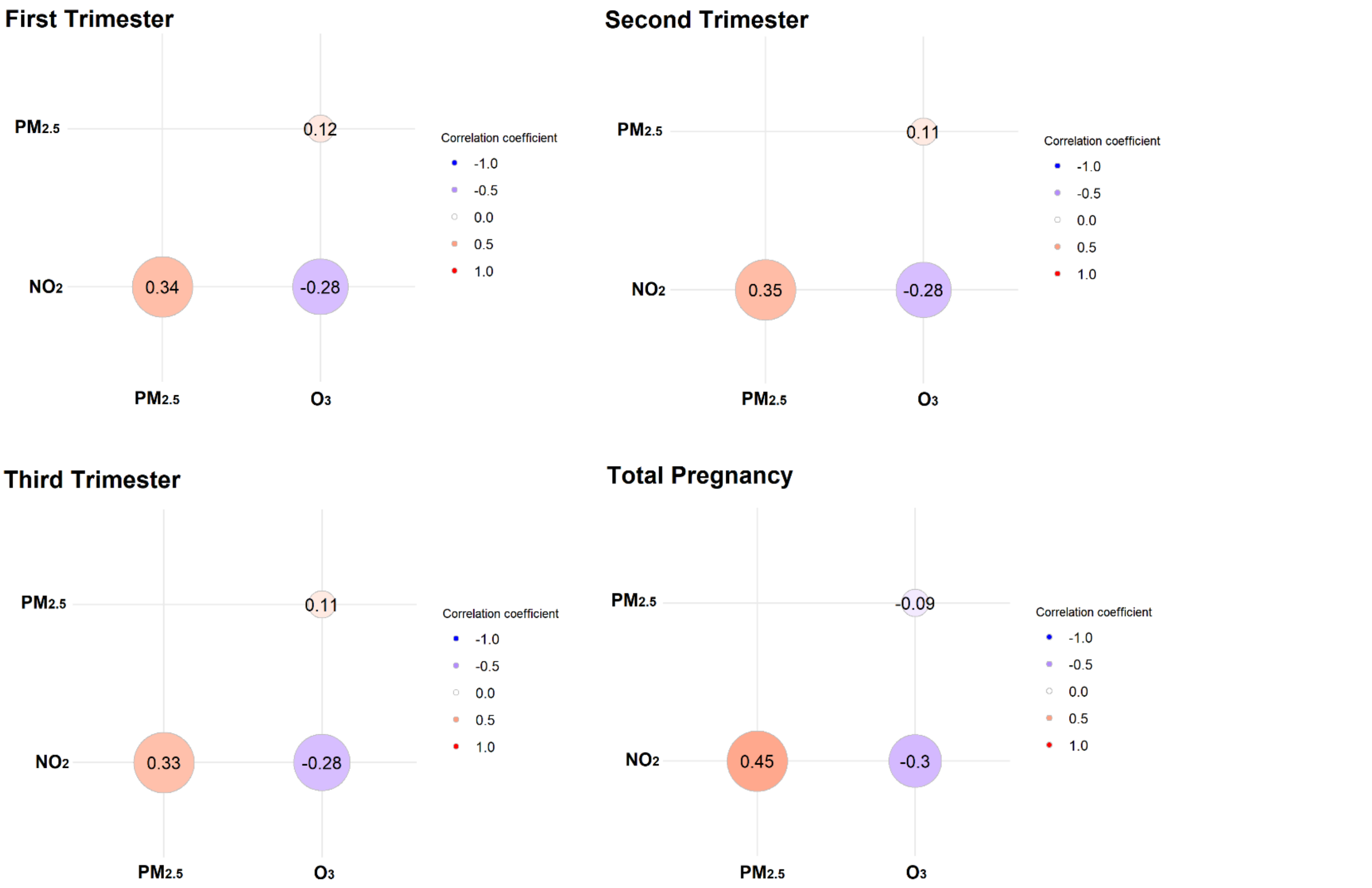

Figure S2. The correlation matrix of different exposure windows of 3 pollutants for preterm birth cohort (N=596,926), Kansas, U.S.A., with an estimated date of conception from January 1st, 2000, to December 31st, 2015.

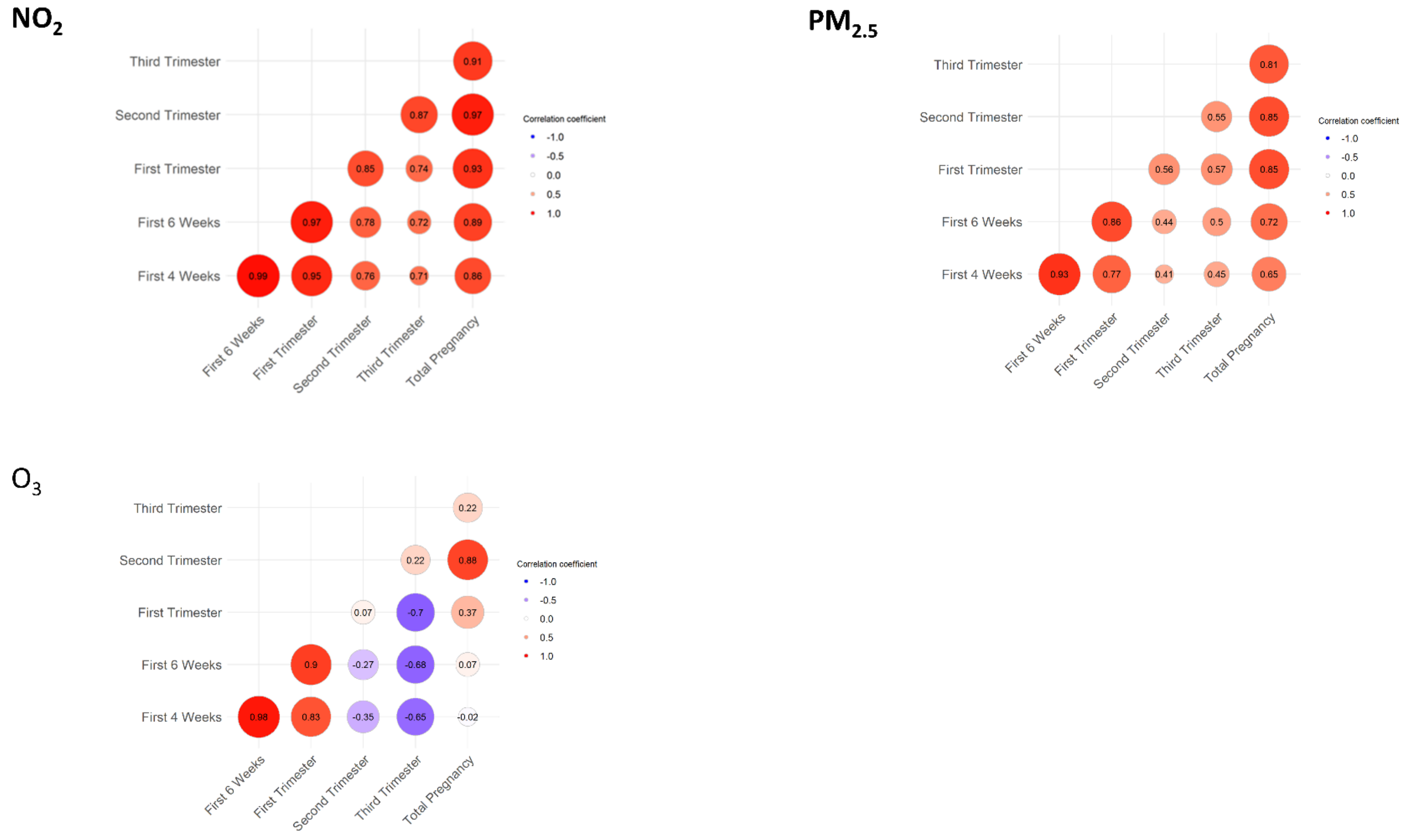

Supplement: Supplementary file 1 — Supplementary Information. [file 41598_2023_48329_MOESM1_ESM.pdf]
